# Development and GBS-genotyping of introgression lines (ILs) using two wild species of rice, *O. meridionalis* and *O. rufipogon,* in a common recurrent parent, *O.* *sativa* cv. Curinga

**DOI:** 10.1007/s11032-015-0276-7

**Published:** 2015-02-14

**Authors:** Juan D. Arbelaez, Laura T. Moreno, Namrata Singh, Chih-Wei Tung, Lyza G. Maron, Yolima Ospina, César P. Martinez, Cécile Grenier, Mathias Lorieux, Susan McCouch

**Affiliations:** 1 Department of Plant Breeding and Genetics, Cornell University, 162 Emerson Hall, Ithaca, NY 14853-1901 USA; 2 School of Botany, The University of Melbourne, Parkville, VIC 3010 Australia; 3 Department of Agronomy, National Taiwan University, No. 1, Section 4, Roosevelt Road, Taipei, 106 Taiwan; 4 Rice Program, International Center for Tropical Agriculture (CIAT), AA6713 Cali, Colombia; 5 CIRAD-AGAP 34394, Montpellier Cedex 5, France; 6 DIADE Research Unit, Institut de Recherche Pour le Développement, 34394 Montpellier Cedex 5, France; 7 Rice Genetics and Genomics Laboratory, International Center for Tropical Agriculture (CIAT), AA6713 Cali, Colombia

**Keywords:** Rice, *Oryza sativa*, Crop wild relatives, Chromosome segment substitution lines (CSSLs), Allele discovery, Marker-assisted selection (MAS), Genotyping by sequencing (GBS)

## Abstract

Two populations of interspecific introgression lines (ILs) in a common recurrent parent were developed for use in pre-breeding and QTL mapping. The ILs were derived from crosses between cv Curinga, a *tropical japonica* upland cultivar, and two different wild donors, *Oryza meridionalis* Ng. accession (W2112) and *Oryza rufipogon* Griff. accession (IRGC 105491). The lines were genotyped using genotyping-by-sequencing (GBS) and SSRs. The 32 Curinga/*O. meridionalis* ILs contain 76.73 % of the donor genome in individual introgressed segments, and each line has an average of 94.9 % recurrent parent genome. The 48 Curinga/*O. rufipogon* ILs collectively contain 97.6 % of the donor genome with an average of 89.9 % recurrent parent genome per line. To confirm that these populations were segregating for traits of interest, they were phenotyped for pericarp color in the greenhouse and for four agronomic traits—days to flowering, plant height, number of tillers, and number of panicles—in an upland field environment. Seeds from these IL libraries and the accompanying GBS datasets are publicly available and represent valuable genetic resources for exploring the genetics and breeding potential of rice wild relatives.

**Electronic supplementary material:**

The online version of this article (doi:10.1007/s11032-015-0276-7) contains supplementary material, which is available to authorized users.

## Introduction

Asian rice (*Oryza sativa* L.) is the staple crop for 3 billion people around the world (Food and Agricultural Organization 2003; Bouman et al. 2007). To meet the growing demand for food driven by population growth and economic development, global rice production must double by 2050 (Ray et al. 2013). Much of this increase is expected to come from new crop varieties that are high yielding, resource-use efficient and resistant to diseases, insects and abiotic stresses, problems that are exacerbated by climate volatility (Godfray et al. 2010; Tester and Langridge 2010). Genetic variation is the raw material used by plant breeders to improve traits and characteristics of interest for producers and consumers (Asíns 2002). While intensive breeding for modern, high-yielding varieties has globally reduced the genetic diversity of crops in farmers’ fields, there is still abundant natural variation in landraces and crop wild relatives conserved in national and international gene banks that can be tapped to accelerate crop improvement for the future (Asano et al. 2011; Gao et al. 2006; Lorieux et al. 2004; McCouch et al. 2013).

Wild relatives of rice are of particular interest as donors of genetic variation because they contain a variety of traits and trait complexes that were eliminated from the cultivated gene pools during the early phases of crop domestication. Wild genetic diversity is most effectively tapped for crop improvement through the creation of interspecific populations via backcrossing with a well-adapted and productive cultivar—a practice that is often termed ‘pre-breeding’. Yet this process is labor and time-consuming and typically fraught with difficulties due to incompatibility barriers, limited recombination, and linkage drag, all of which limit the ease of identifying, transferring, and utilizing beneficial wild alleles in crop improvement (Lorieux et al. 2004; Brar and Khush 1997; Tanksley and McCouch 1997).

The use of DNA-marker technology has greatly accelerated the development of pre-breeding populations and facilitated the targeted introduction of useful variation into elite breeding backgrounds. It has also enabled dissection of quantitative trait locus (QTL) and the discovery of wild alleles underlying traits of agronomic importance (Wang et al. 1992; Song et al. 1995; Zamir 2001; Collard and Mackill 2008; Chin et al. 2011; Periyannan et al. 2013; Saintenac et al. 2013). Recent developments in next generation sequencing, including the use of reduced representation libraries in genotyping by sequencing (GBS), have further reduced the cost and increased the resolution and throughput of trait mapping and marker-assisted breeding, making it faster and easier to genetically characterize and select lines with favorable wild alleles for use in breeding.

Advanced backcross populations, such as near-isogenic lines (NILs) (Inukai et al. 1996; Takahashi et al. 2001; Maas et al. 2010; Imai et al. 2013), Introgression Lines (ILs) (Thomson et al. 2003, 2006; Tian et al. 2006; Gutierrez et al. 2010; Ogawa et al. 2014), and chromosome segment substitution lines (CSSLs) (Kubo et al. 2002; Ebitani et al. 2005; Ando et al. 2008; Ali et al. 2010; Xu et al. 2010) are commonly used when working with intra and interspecific crosses of rice. In these materials, small chromosomal segments containing alleles from a wild donor parent are systematically introduced into a cultivated genetic background using marker-assisted selection (MAS). Each line contains only one or few well-defined introgressions in an adapted, recurrent parent (RP) background. This is advantageous so the effect of individual wild alleles can be determined in the genetic background of a commercially acceptable variety (Tanksley and Nelson 1996; Kubo et al. 2002; Ebitani et al. 2005; Kanbe et al. 2008; Ali et al. 2010; Gutierrez et al. 2010). In addition, when fixed lines are used for evaluation, identical genotypes can be evaluated repeatedly in different seasons and environments to improve the accuracy of QTL detection (Liu et al. 2008). ILs can also be used to rapidly develop secondary F_2_ populations for fine mapping and positional cloning of interesting genes and QTL (Yano et al. 2000).

The two wild species used as donors in this study, *O. meridionalis* Ng. and *O. rufipogon* Griff., both carry the AA genome and are cross-compatible, but are estimated to have diverged approximately 2 million years ago (Park et al. 2003; Ren et al. 2003; Zhu and Ge 2005). *O. meridionalis* is considered to be the basal lineage of the AA genome species and is the most divergent with respect to cultivated Asian rice, *O. sativa* (Zhu et al. 2014). In contrast, *O. rufipogon* is considered to be the immediate ancestor of *O. sativa. O. meridionalis* is native to Australia and some regions of West Papua and Indonesia (Ng et al. 1981; Lu and Silitonga 1999), while *O. rufipogon* is found throughout tropical mainland and South East Asia.

In this study, we apply marker-assisted backcrossing (MABC) to develop two sets of advanced backcross ILs by crossing an *O. meridionalis* accession, W2112, hereafter referred to as *MER*, and an *O. rufipogon* accession, IRGC 105491, hereafter referred to as *RUF*, with a *tropical japonica* cultivar from Brazil, cv Curinga, hereafter referred to as *CUR* (de Morais et al. 2005). Few attempts to develop pre-breeding materials utilizing *MER* as a donor have been reported (Yoshimura et al. 2010; Doi et al. 2003), while *RUF* has been used in several previous QTL and pre-breeding studies and is known to carry favorable alleles for yield and yield components (Xiao et al. 1998; Moncada et al. 2001; Thomson et al. 2003; Septiningsih et al. 2003; Marri et al. 2005; McCouch et al. 2007; Imai et al. 2013).

We demonstrated the usefulness and versatility of these ILs for genetic mapping and as pre-breeding germplasm by evaluating their segregation for one Mendelian trait, red pericarp, and four quantitative traits, flowering time, plant height, tiller number and panicle number, based on greenhouse and field studies. Red pericarp is a highly heritable trait, regulated by the *RC* gene (Sweeney et al. 2006). A 14-bp deletion in the sixth exon of *RC* is the most common mutation leading to white pericarp. The functional version of *RC* is found in virtually all wild rice and confers both red grain color and seed dormancy. While both traits were selected against during rice domestication, some degree of dormancy is still important in white pericarp cultivars where pre-harvest sprouting may otherwise become a problem (Gu et al. 2011). More recently, cultivated red rice varieties, such as the Buthanece Red, Thai Red Cargo and the French Camargue Red Rice, have sought to bring back the health benefits associated with pigmented pericarp, while avoiding the problems associated with excessive degrees of dormancy. These varieties have the potential to be commercialized in the rice specialty varieties market.

We also evaluated the collection of *CUR/RUF* introgression lines for four agronomic traits, flowering time, plant height, tiller number, and panicle number, under upland field conditions. Rice grown in the upland farming system constitutes only 12 % of the total area planted on a global basis, but in Latin America, it accounts for 45 % of rice area, with 69.6 % of rice in Brazil and 23 % in Colombia grown as a dry land crop (Moncada et al. 2001). Many upland soils are highly acidic, making it necessary to breed for acid soil tolerance as well as yield potential for these systems. We were interested to determine whether particular *O. rufipogon*-derived alleles might confer positive effects in the *CUR* genetic background for the four traits of interest, and to determine the effect of the introgressions in soils that were naturally acidic (pH < 5.0) and in soils that were limed (pH > 5.0).

## Materials and methods

### Plant materials

The common recurrent parent Curinga (*O. sativa ssp. tropical japonica*) (*CUR*) is a commercial rice variety released in 2005, developed by the Empresa Brasileira de Pesquisa Agropecuária (EMBRAPA, Goiania, Brazil) (de Morais et al. 2005). It is a semi-early maturing, drought-tolerant cultivar with an average yield under upland conditions of 4,465 kg/ha. This cultivar is characterized by long fine grains with good whole kernel yields, resistance to rice blast and leaf scald, tolerance to acid soils and drought conditions (de Morais et al. 2005). The wild donor parent, *O. meridionalis* Ng, acc. W2112 (*MER*) (Oryzabase: http://www.shigen.nig.ac.jp/rice/oryzabaseV4/) was collected in Cooktown, Australia, by Gérard Second, IRD, Montpellier. It shows strong dormancy, seed shattering, photoperiod sensitivity and good tillering proliferation (results not shown). The wild donor *O. rufipogon* Griff. acc. IRGC 105491 (*RUF*) (International Rice Research Institute, IRRI; http://www.irgcis.irri.org:81/grc/IRGCISHome.html) was collected in Kelantan, Malaysia. This wild accession shows high seedling vigor, good germination rates, good tiller proliferation, and upright grow habit (results not shown). Hereafter, the Curinga × *O. meridionalis* population will be referred to as *CUR/MER* and the Curinga × *O. rufipogon* population will be referred to as *CUR/RUF*.

### Linkage map construction

Two genetic maps were developed using 110 BC_1_F_1_ *CUR/MER* plants genotyped with 122 simple sequence repeat markers (SSR), and 80 BC_1_F_1_ *CUR/RUF* plants genotyped with 131 SSR markers (Orjuela et al. 2009). The genetic linkage maps were generated using the software MapDisto v.1.7.5 (Lorieux 2012; http://mapdisto.free.fr/MapDisto/). Markers were placed into linkage groups using a logarithm of odds (LOD) probability that two markers are linked versus non-linked of 3.0 and a maximum recombination fraction (*r*_max_) of 0.3. Marker order in linkage groups was determined using a combination of the ‘Order,’ ‘Ripple’ and ‘Check inversions’ commands. Data errors were then corrected using the ‘Color Genotypes’ module in MapDisto. Recombination fractions were transformed to estimate map distance using the Kosambi mapping function (Kosambi 1943; Lorieux 2012). A Chi-square test (*χ* ^2^) was carried out for each marker to test deviations of genotypic classes from the expected Mendelian inheritance ratios of 1:1 (*p* < 0.01). The ‘Compare maps’ command was used to graphically compare colinearity between the generated linkage maps with physical distances from the Michigan State University (MSU) version 7 Rice Genome assembly (http://rice.plantbiology.msu.edu/).

### Development of introgression lines (ILs)

Using marker-assisted backcrossing (MABC) two IL libraries were developed. Fixed lines were selected after three rounds of MABC and one generation of double haploidization (DH) using anther culture (Lentini et al. 1995) (Online resource 1). A set of 32 and 48 fixed lines were selected to comprise the *CUR/MER* and *CUR/RUF* populations, respectively. Between each crossing cycle (Sarkarung 1991), SSRs (McCouch et al. 2002, http://www.gramene.org) comprising the Universal Core Genetic Map (UCGM) (Orjuela et al. 2009) and transposable-element-based (TE) indel markers were used to perform foreground and background selection (Hospital et al. 1997). In total, 32 *CUR/MER* lines were genotyped with 122 SSRs and nine TE-based markers and 48 *CUR/RUF* lines were screened with 131 SSRs as the basis for selecting the IL libraries. Separate sets of markers were used on the two populations due to differences in polymorphism between the parents. Genotypic selection was implemented using CSSL Finder v. 0.9 (Lorieux 2005; http://mapdisto.free.fr/CSSLFinder/) which enables selecting the minimum set of lines to cover the entire donor genome with a desired number and size of included introgressions, small overlaps between consecutive introgressed fragments, and minimal background genome recovery. Genotyping by sequencing (GBS, Elshire et al. 2011) was conducted in the final generation (BC_3_F_1_-DH).

### Double haploid protocol

DH plants were generated as described by Lentini et al. (1995). Seeds were sown in germination trays and grown in the greenhouse for 20–25 days. Seedlings were then transplanted to the field and space-planted at 30 × 40 cm between plants and rows, and grown under standard flooded (paddy) conditions. At booting stage (~60–70 days after sowing, depending on the genotype), when only 5–8 cm of the emerging panicle (boot) was visible above the sheath, 3–5 immature panicles (still wrapped in the sheath) were harvested from each plant and kept in polyethylene bags in the dark for 7 days at 10 ± 2 °C before culturing.

Panicles were surfaced-sterilized using sterile distilled water and 70 % ethanol. Spikelets from the second third of the panicle were then removed from the sheath leaf, soaked for 3 min in 10 % commercial bleach (5.25 % NaOCl), mixed with three drops of Tween 80, and rinsed with sterile distilled water. Each flower was cut at the base, at the anther filament, and grouped into clusters of 10–15 cut flowers. Each cluster was picked with forceps, and the anthers were released by tapping the forceps on the edge of the culture jar (7 cm high × 4 cm diameter). For each jar, ~250 anthers per 10 ml of liquid callus induction medium were cultured. Jars were sealed with Magenta-B caps and kept in the dark at 25 ± 1 °C during 4–6 weeks. Ten embryogenic calli of approximately 1–2 mm in diameter were transferred onto 60 ml regeneration medium contained in 12 cm high × 9 cm diameter glass jars. Jars were sealed with a plastic cap and placed under indirect light for one week and then moved to 80 µE/m^2^/s and 16 h photoperiod. Temperature was kept steady at 25 ± 1 °C. After 4–6 weeks, green regenerated plants with fully developed roots were transplanted to sterile soil in the greenhouse for 3–4 weeks and then moved to the field. Haploid, DH or diploid, and polyploid plants were identified by evaluating plant morphology and fertility. The type-A basal callus induction media, and the components and quantities of plant regeneration media used to generate the DH plants were as described by Lentini et al. (1995).

### SSR marker analysis

Fresh leaf tissue was collected during each generation, and genomic DNA was extracted using a CTAB protocol described by Romero et al. (2014). DNA concentration and quality were evaluated on 0.8 % agarose gels stained with ethidium bromide. The two subsets of SSR from the UCGM set were used in genotyping. Polymerase chain reactions (PCR) were performed using 20 ng of DNA as template, 100 mM Tris–HCL, 500 mM KCL, 0.1 % Triton X-100, 2 ng/µl MgCl_2_ (25 µm), 0.3 µM of each dNTP and primer, and 1 U of *Taq* DNA polymerase. The reactants were initially denaturated at 94 °C for 3 min, followed by 30 cycles at 94 °C for 30 s, 30 cycles at 50–67 °C (depending on annealing temperature of each primer pair) for 45 s, 72 °C for 1 min, and a final extension at 72 °C for 5 min. PCR amplified fragments were analyzed using 4 % agarose gels stained with ethidium bromide or 6 % denaturing acrylamide gels stained with silver staining (Panaud et al. 1996).

### GBS library preparation and data analysis

Total genomic DNA was extracted from each IL and parents using the Qiagen DNeasy kit (http://www.qiagen.com/). 96-plex libraries were prepared according to Elshire et al. (2011). A custom-designed pipeline described by Spindel et al. (2013) that combines the built-in TASSEL (Bradbury et al. 2007; http://www.maizegenetics.net/#!tassel/c17q9) SNP caller and the sequence aligner algorithm from PANATI with the genotype imputation algorithm GBS-PLAID-2 was used for data analysis.

### Custom Infinium 6K SNP assay and data analysis

GBS single-nucleotide polymorphism (SNP) calls were confirmed using a custom-designed Infinium HD SNP Assay (M. Wright, Cornell University, pers. comm.; http://www.illumina.com) that detected 1,092 segregating SNPs in the *CUR/MER* and 1,769 in the *CUR/RUF* populations using the same DNA extracted for GBS assays. The 6K Infinium assay required 750 ng—1 µg of high molecular weight DNA. The DNA was isothermally amplified overnight and a controlled enzyme process fragmented the amplified product. The fragmented DNA was alcohol precipitated and resuspended for hybridization. The BeadChip was prepared for hybridization in a capillary flow-through chamber to which the amplified-fragmented DNA samples was applied and incubated overnight. During the hybridization step, the DNA annealed to locus-specific SNP markers (50-mers) and the allele specificity was conferred by base extension. The products were fluorescently labeled and intensity was detected and recorded by the Illumina BeadArray reader. Illumina’s genome studio software was used for analyzing the genotype calls. Graphical genotypes were drawn using CSSL Finder v.0.9 (Lorieux 2005).

### Genetic analysis of pericarp color

Five grains from each of the parents, *CUR, MER, RUF,* and each IL were de-hulled, photographed and visually scored for pericarp color. Each phenotyped seed was coded 1 = pigmented pericarp, or 0 = white pericarp. Two ILs from the *CUR/MER* and 7 ILs from the *CUR/RUF* populations carried the red pericarp and were used to determine whether the *RC* gene was responsible for the trait. A functional indel marker designed by Sweeney et al. (2006) was used to amplify the 14-bp insertion/deletion in the *RC* gene (LOC_Os07g11020.1) responsible for white pericarp (forward primer: CTTGCCAGTTTCAGAGAAATCA; reverse primer: CTCTTTCAGCACATGGTTGG). PCR products were amplified from the parents, the 9 ILs with red pericarp, 8 ILs with white pericarp (selected at random from the 71 white seeded ILs), and control white-pericarp varieties, IR64 (ssp *indica*) and Azucena (ssp *tropical japonica*). PCR products were visualized using a 4 % agarose gel stained with SYBR^®^ Safe (http://www.lifetechnologies.com/), as described by Sweeney et al. (2007).

### Upland field evaluation of *CUR/RUF* ILs

The recurrent parent, *CUR*, 48 ILs from *CUR/RUF* population, and 15 elite local checks from the CIAT/CIRAD (International Center for Tropical Agriculture/Centre de Cooperation Internationale en Research Agronomique) upland rice program were evaluated under upland conditions using two different agronomic practices; natural soil conditions (pH < 5.0) and limed soil conditions (pH > 5.0), at the experimental field station ‘La Libertad ICA’ (latitude 4°3′40. 63″N, longitude 73°27′46. 25″W) in Meta, Colombia during Summer 2012. (Note: the *RUF* donor parent could not be grown in the field due to its classification as a noxious weed. The *CUR/MER* population was not evaluated in the field because seeds were not available at that time) An alpha lattice design with two replications was used to evaluate the 64 lines distributed in eight blocks, under two treatments: (1) acid soil (pH levels < 5 and aluminum saturation over 75 %), occur under natural conditions in Meta, and (2) lime treated soil, where soil was amended with 3 tons/ha of calcium carbonate (CaCO_3_) before planting (Online Resource 2a). Planting was implemented using a six-row planter with a sowing density of 1 g of seed per linear meter. The plot size was 3 m wide with two rows per plot, spaced 26 cm between rows. Fertilizer was applied in two stages. Before planting 80 kg/ha of DAP (NH_4_)_2_ HPO_4_, 80 kg/ha of KCl (K_2_O), 12 kg/ha of Zn, and 75 kg/ha of kieserite (MgSO_4_·H_2_O) were applied. After planting one application of 40 kg/ha of KCl and three applications of 26.6 kg/ha of urea were added. Weed control was done by applying a post-emergence herbicide, Butaclor 3 L/ha, and Bentazol at 3 L/ha plus manual weeding. The fungicide Bim was applied throughout the vegetative cycle as a preventive disease-control measure.

For phenotyping, one linear meter around the center of each plot was marked and plants within that area were harvested. Four agronomic traits: flowering time, plant height, number of tillers, and number of panicles, were evaluated under both treatments. ‘Flowering time’ was scored as the number of days from planting until 50 % of the plants in the plot have emerged panicles. ‘Plant height’ was measured using a ruler, as the average length in centimeters from five random tillers in the linear meter from the base of the plant to the tip of the panicle. ‘Number of tillers’ was defined as the number of tillers from one linear meter harvested from the middle of the plot. ‘Number of panicles’ was defined as the number of panicles from one linear meter harvested from the middle of the plot.

Broad-sense heritability for each trait was estimated using the formula $H^{2} = \delta_{G}^{2} /\left(\delta_{G}^{2} + \left( {\frac{{\delta_{\text{GE}}^{2} }}{e}} \right) + \left( {\frac{{\delta_{e}^{2} }}{re}} \right)\right)$ (Hallauer et al. 2010) where *e* and *r* are the numbers of environments and replications per environment.

### Statistical analysis

To detect significant differences between the recurrent parent *CUR* and the ILs, a post hoc Dunnett’s pairwise multiple comparison test (Dunnett 1980) with a significant level of 0.01 and using *CUR* as control was performed for each trait. Because most ILs carry more than one segment from the donor parent, a standard single-marker linear regression analysis (SMLRA) was used to identify markers significantly associated with pericarp color, and a stepwise regression analysis (Li et al. 2007) was implemented for flowering time, plant height, number of tillers and number of panicles using the software iciMapping v.3.2 (http://www.isbreeding.net/software/?type=detail&id=13). A likelihood ratio test based on linear regression was used to estimate LOD scores from the *p* values. A permutation test using 1,000 permutations (Doerge and Churchill 1996) was used to determine the experiment-wise significance threshold at a 0.05 level of significance.

## Results

### Development of introgression lines

**Fig. 1:**
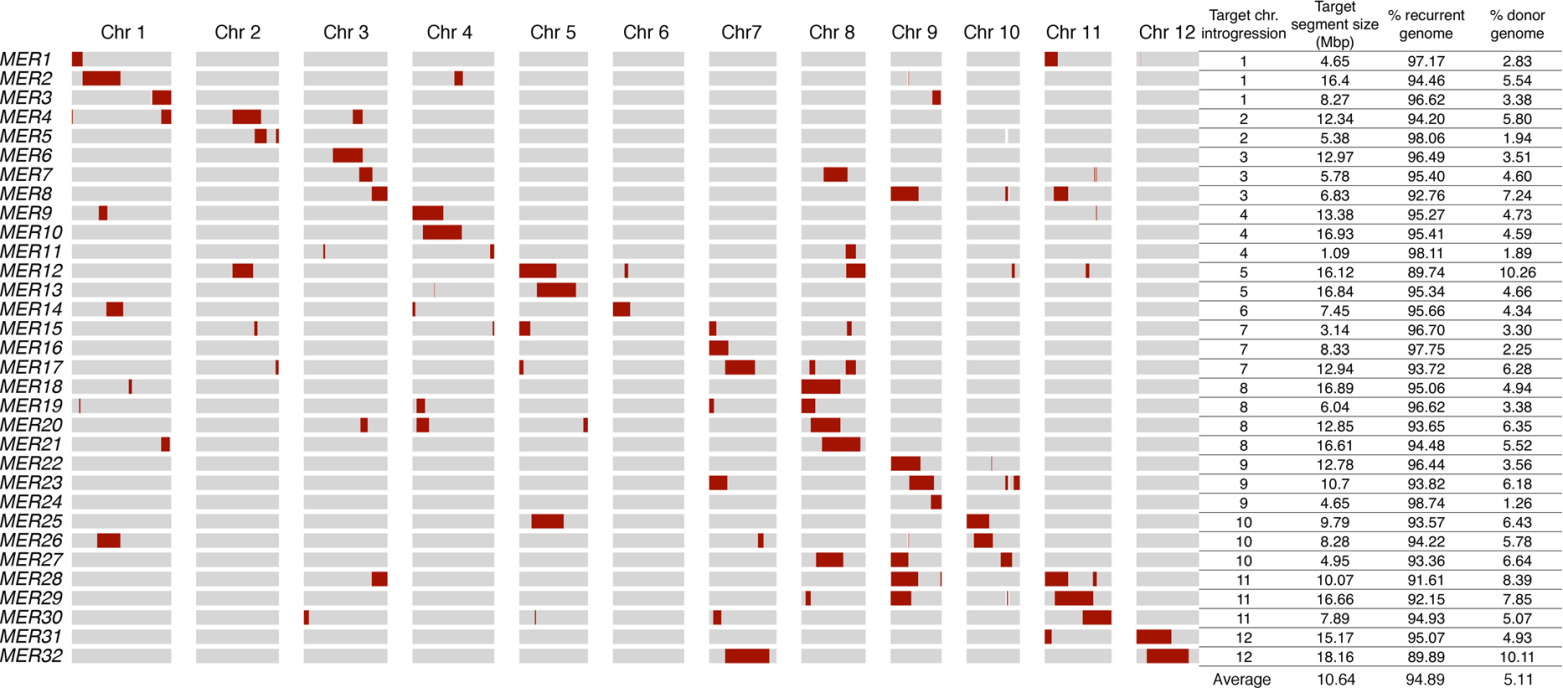
Graphical genotypes, distribution of target and non-target introgressions, and genotypic statistics for 32 *CUR/MER* ILs. Graphic representation of the genotypic make-up for the recurrent parent *CUR* (*gray*), the donor parent *MER* (*red*) in the 32 ILs (*MER1*-*MER32*) from the *bottom* to the *top* of the *graph* across each of the 12 chromosomes. The chromosome where the target introgression is located, its base-pair size, number of donor segments and percentage of recurrent and donor genome are listed next to each IL. (Color figure online)

**Fig. 2:**
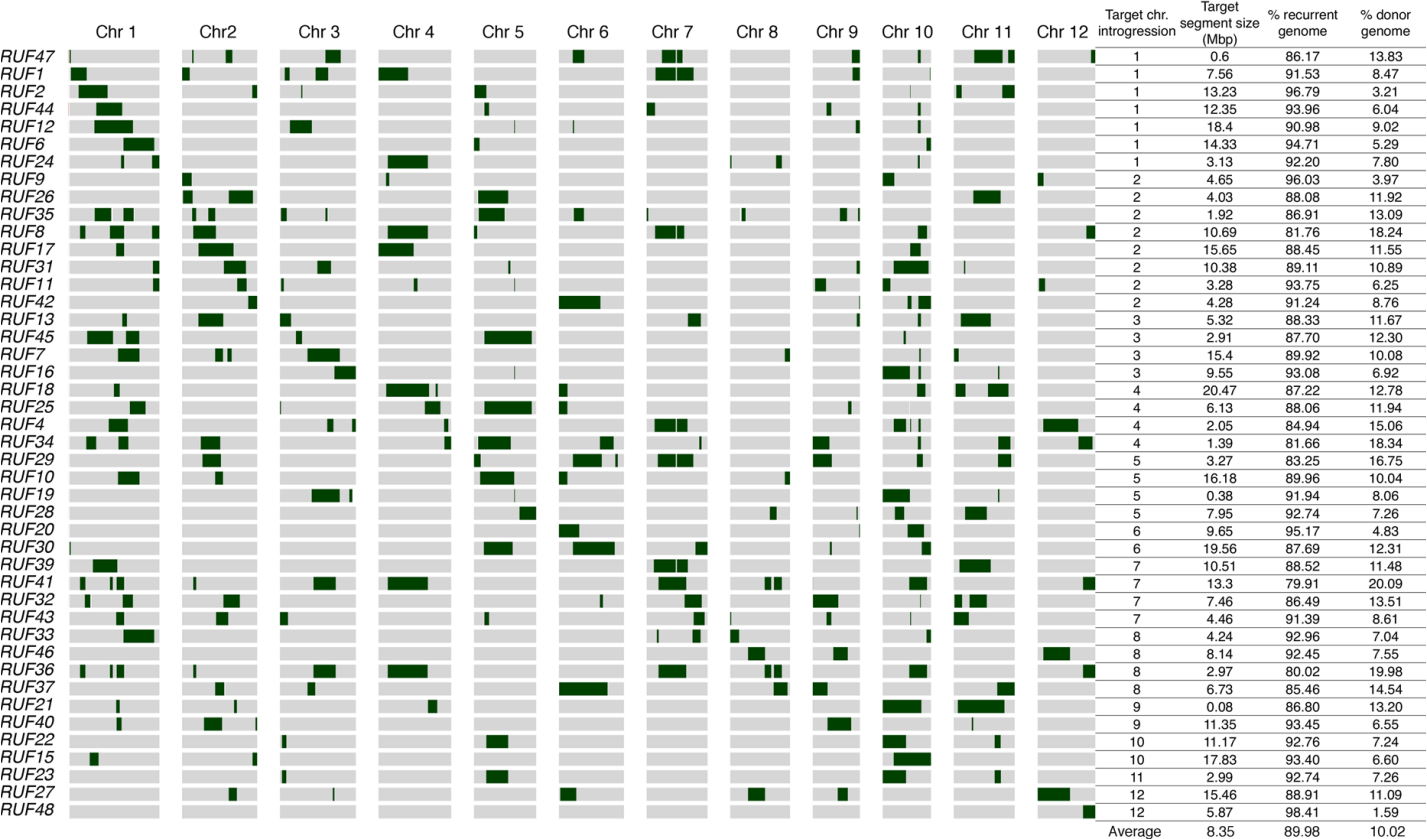
Graphical genotypes and distribution of target and non-target introgressions for 40 *CUR/RUF* ILs. Graphic representation of the genotypic make-up for the recurrent parent *CUR* (*gray*), the donor parent *RUF* (*green*) in 40 ILs (*RUF1*-*RUF48*) from the *bottom* to the *top* of the *graph* across each of the 12 chromosomes. The chromosome where the target introgression is located, its base-pair size, number of donor segments and percentage of recurrent and donor genome are listed next to each IL. (Color figure online)

A marker-assisted backcrossing strategy (MABC) was implemented to develop the two IL libraries. Fixed lines were selected after three rounds of MABC and one generation of double haploidization using anther culture (Lentini et al. 1995) (Online resource 1). A set of 32 and 48 fixed lines were selected to comprise the *CUR/MER* and *CUR/RUF* introgression libraries, respectively (Figs. 1, 2). Together, the lines comprising each library contain the majority of the donor genome introgressed as small overlapping chromosome fragments in the *CUR* genetic background.

A linkage map for each interspecific cross was generated from SSR segregation data in the BC_1_F_1_ generation to confirm the colinearity of the genomes in *O. sativa* and the distantly related wild donor genomes. The SSR linkage maps also served as the basis for genotypic selection during the first three backcross-generations of IL population development (Online Resource 1). The genetic maps had total distances of 2,005.25 cM (*CUR/MER*) and 1,797.57 cM (*CUR/RUF*) (Online Resource 3a and 3b). The average distance between markers was 16.41 cM in *CUR/MER* and 13.95 cM in *CUR/RUF* (Online Resource 4a and 4b). When the positions of markers on the respective genetic maps were compared using pseudo-centimorgans (1 cM ~ 240 Kbp), we observed complete colinearity in the two populations (Online Resource 4a and 4b). A Chi-square test for skewed segregation of genotypic classes showed strong deviation (*p* < 0.01) for 18 markers on six chromosomes in the *CUR/MER* population (Online Resource 5a and 5b), but no skewed segregation rations were observed in the *CUR/RUF* population.

**Fig. 3:**
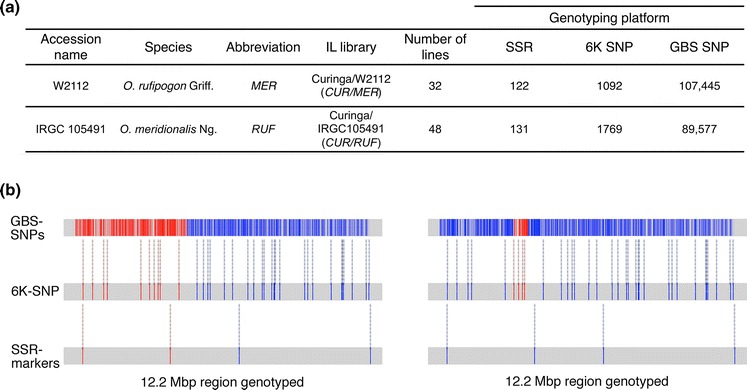
**a** Polymorphic markers between the recurrent parent *CUR* and the two donor parents *MER* and *RUF*, using SSR markers, and SNP-markers from 6K Infinium platform and GBS platform. **b** Genotyping-platforms comparison. Illustration of a 12.2 Mbp region in chromosome 1 genotyped in two *CUR/MER* lines, *MER3* (on the *left*) and *MER19* (on the *right*) using SSR markers, a 6K-SNP Infinium chip and GBS. Respectively for each platform 4, 35 and 4,079 markers segregate. The *MER* alleles are *color*-*coded red*, and the *CUR* alleles are *color*-*coded blue*. Comparisons between platforms are determined by *dashed lines*. (Color figure online)

In the BC_3_F_1_DH generation, the two libraries were genotyped using GBS to increase marker density, improve estimation of each recombination break point and to detect donor introgressions that had gone undetected using the sparse coverage provided by the SSR and indel markers (Fig. 3a, b). The number of polymorphic markers identified between *CUR* and the two wild donors is summarized in Fig. 3a for the SSR/indels, the 6K SNPs and the GBS markers. Using the 6K SNP assay, marker density increased over tenfold, from an average of 16.41 cM/marker using SSRs to 1.70 cM/marker in the *CUR/MER* and from an average of 13.95 cM/marker to 1.03 cM/marker in the *CUR/RUF* population. Using GBS, marker density increased again, an estimated 50-fold, from an average of 1.70 cM/marker using the 6K assay to 0.02 cM/marker in the *CUR/MER* and from an average of 1.03 cM/marker using the 6K assay to 0.024 cM/marker in the *CUR/RUF* population. In practical terms, this increase in resolution allowed us to detect additional introgressions that had been missed using SSRs and to better define the size and genome positions of both target and background introgressions in all the ILs as illustrated in Fig. 3b.

In the *CUR/MER* IL library, donor introgressions cover 76.73 % of the *MER* genome. The missing portion of the donor genome (23.27 %) is distributed in 13 regions across nine different chromosomes (Online Resource 6a). For the *CUR/RUF*, the donor introgressions cover 97.6 % of the *RUF* genome. The small missing portion of the donor genome (2.4 %) is distributed in 11 regions across four different chromosomes (Online Resource 6b). The location of each targeted introgressed donor segment, number of donor introgressions and percentage of background donor genome for each ILs are summarized in Figs. 1, 2.

### Phenotypic evaluation

To demonstrate the utility of the IL libraries for mapping and as pre-breeding materials, we evaluated both populations for a simply inherited trait (pericarp color) in the greenhouse, and the *CUR/RUF* population for four quantitatively inherited traits under upland conditions in the field.

#### Pericarp color

**Fig. 4:**
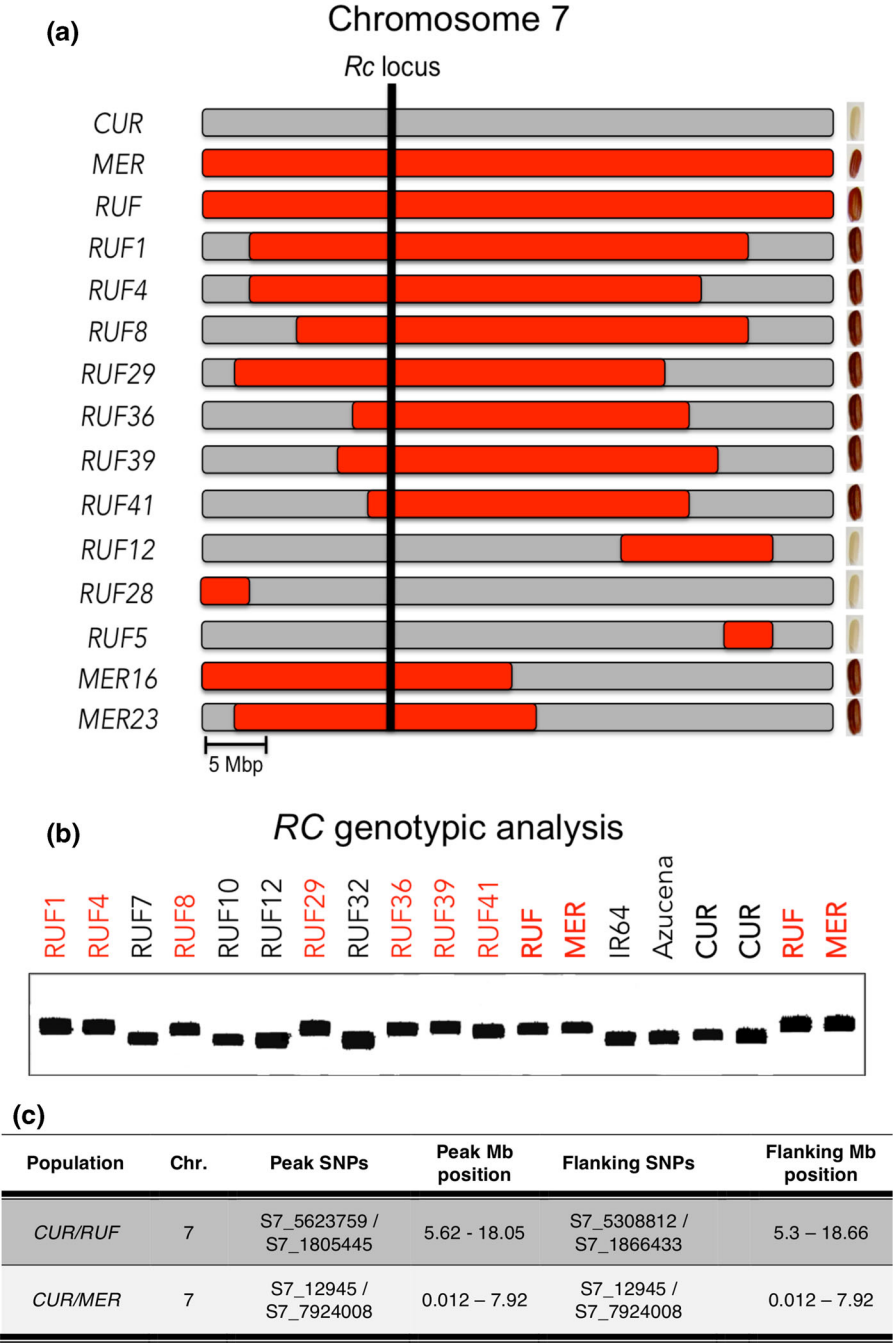
Chromosome 7 donor introgressions **a** Chromosome 7 zoom-in. Genotypic introgression of 12 different IL and parents in chromosome 7, the recurrent genome is colored in *gray* and the donor genome in *red*. The phenotype of each IL is shown in the *right border* **b** PCR products to detect the functional 14-bp indel marker in the *Rc* locus (Sweeney et al. 2006) from seven colored-pericarp ILs, and four *white colored*-pericarp, the parents *CUR*, *RUF*, *MER* and two controls lines IR64 and Azucena. **c** Summary of significant regions associated with pericarp color (LOD > 3.1) using logistic regression (SMA-LR) analysis. (Color figure online)

The wild donor parents, *MER* and *RUF,* both have red pericarp, while *CUR* has a translucent or white pericarp (Online Resource 7). Two ILs from the *CUR/MER* library (*MER16* and *MER23*) and seven from the *CUR/RUF* library (*RUF1*, *RUF4*, *RUF8*, *RUF29*, *RUF36*, *RUF39*, and *RUF41*) were identified with red pericarp color, while all other ILs in both libraries had white pericarp (Fig. 4a, Online Resource 7). When the functional indel marker previously developed by Sweeney et al. (2006) was used to determine which lines carried the wild-type allele and which carried the derived allele at *RC,* both *MER* and *RUF,* as well as the two *CUR/MER* ILs and the seven *CUR/RUF* ILs with red pericarp were confirmed to carried the wild-type (non-deletion) allele (Fig. 4b), while the *CUR* recurrent parent, the eight randomly selected white pericarp ILs and two white-seeded control varieties, IR64 (ssp *indica*) and Azucena (ssp *tropical japonica*), all carried the derived (14-bp deletion), recessive allele (Fig. 4b and Online Resource 7).

The two red *CUR/MER* ILs shared a wild introgression on chromosome 7 in the region containing *RC*, defined by the GBS-SNP markers S7_12945 and S7_7924008 and mapping to the interval 07:12,945–07:7,924,008 bp on the physical map of rice (MSU7.0, Kawahara et al. 2013). To confirm that allelic variation at the *RC* gene was also predictive of pericarp color in the *CUR/RUF* ILs, where lines carry multiple donor introgressions in the genetic background, we conducted a SMLRA and identified seven markers significantly associated with pericarp color (LOD > 3.1). All seven SNPs were located on chromosome 7 between positions 07:5,308,812–07:18,659,022 bp (MSU7.0, Kawahara et al. 2013) (Fig. 4c). The *RC* gene is located at 07:6,061,890–6,068,318 bp and is contained in the overlapping segment shared by these lines (Fig. 4a). In both populations, the functional marker in *RC* is a perfect predictor of pericarp color in our IL libraries.

### Upland field evaluation

When the *CUR/RUF* ILs were evaluated for four agronomic traits in the field, the distribution of ‘plant height,’ ‘tiller number,’ and ‘panicle number’ was normal, while the distribution of ‘days to flowering’ was skewed toward late flowering (Online Resource 2b). ‘Tiller number’ and ‘panicle number’ were strongly correlated (*r* = 0.9) (Online Resource 2b). Broad sense heritability (*H* ^*2*^) estimations showed better repeatability for ‘days to flowering’ (*H* ^2^ = 0.97) and ‘plant height’ (*H* ^2^ = 0.85) than for ‘number of tillers’ (*H* ^2^ = 0.55) and ‘number of panicles’ (*H* ^2^ = 0.51).

**Fig. 5:**
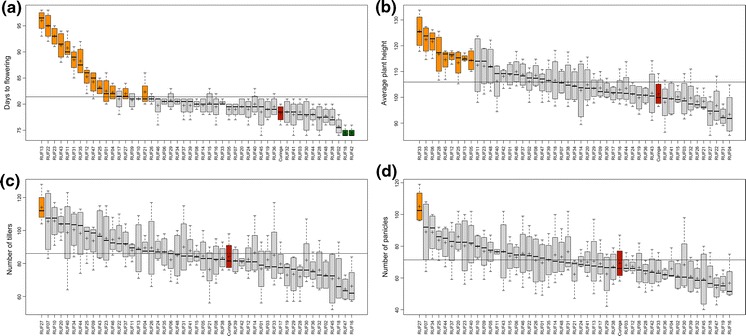
*CUR/RUF* upland field evaluation Boxplots showing the distribution of *CUR/RUF* ILs for four phenotypes. In each, the recurrent parent (*CUR*) is colored red and ILs significantly higher than *CUR* (*p* < 0.05) are *colored orange*, and lower than *CUR* (*p* < 0.05) are *colored green*. **a** ‘Days to flowering.’ **b** ‘Average plant height.’ **c** ‘Number of tillers.’ **d** ‘Number of panicles’. (Color figure online)

Significant differences among IL genotypes (*p* < 0.05) were observed for all traits (Fig. 5). For flowering time, 16 ILs were significantly different from the recurrent parent: two ILs flowered earlier than *CUR*, while 14 ILs flowered later (*p* < 0.05; Fig. 5a). Nine ILs were significantly taller than *CUR* (*p* < 0.05; Fig. 5b). Of particular note, IL *RUF27* had a significantly higher number of both tillers and panicles per linear meter than *CUR* (*p* < 0.05; Fig. 5c, d). This IL warrants further testing to determine its relevance as a potential donor in breeding.

**Fig. 6:**
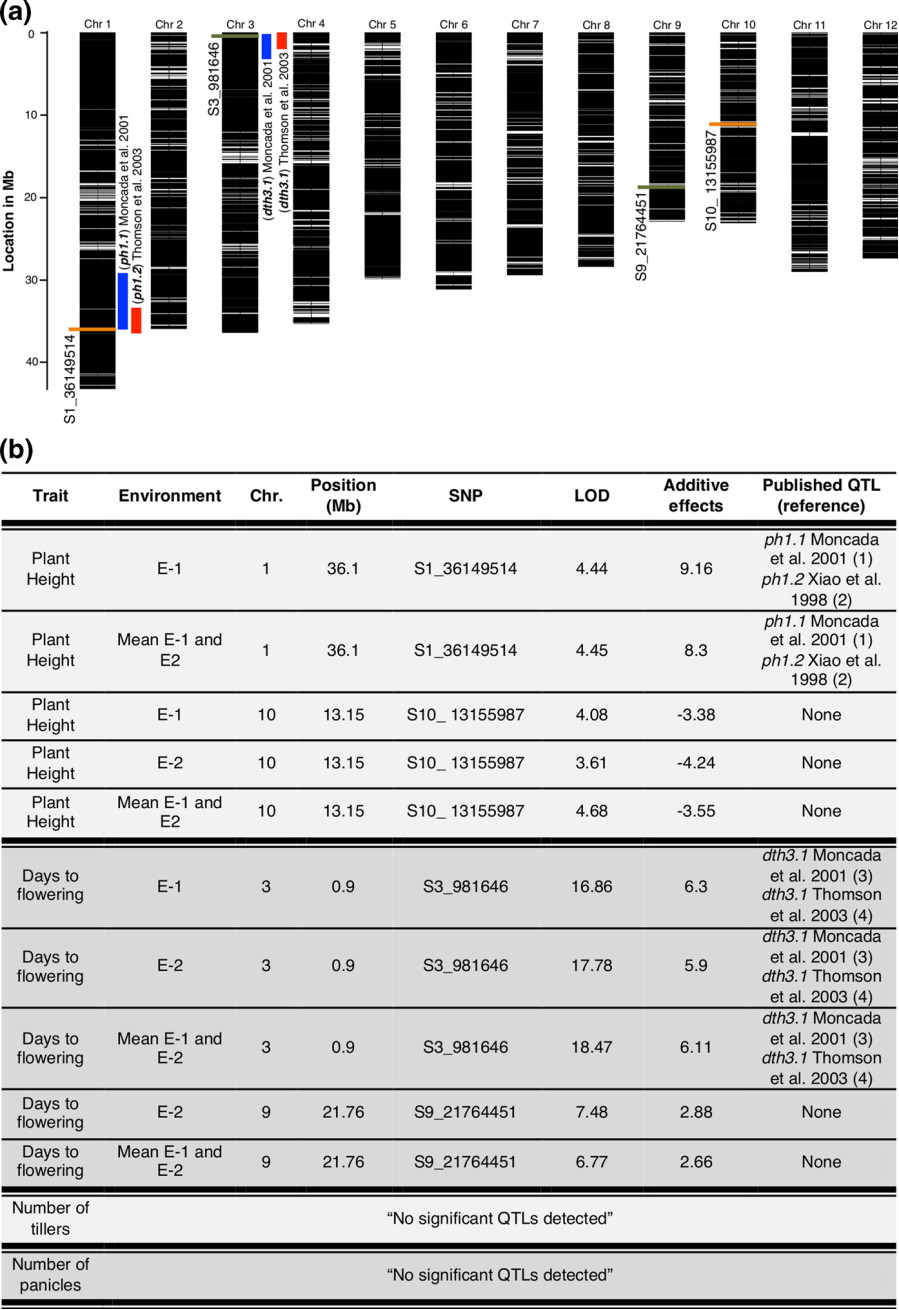
Upland field evaluation summary, **a** GBS SNP coverage across the *CUR/RUF* ILs population (*black lines* in chromosome bars), graphic location of SNPs significantly associated with ‘days to flowering’ (*green lines*) and ‘plant height’ (*orange lines*), and published QTL regions that co-localize with the associated SNPs from this study (*red* and *blue bars*). **b** Summary of significant regions associated with plant height (LOD > 3.5), and days to flowering (LOD > 5) using a stepwise regression single-marker analysis (SR-SMA) for days to flowering and plant height. (Color figure online)

When the performance of ILs was compared between plants grown in naturally occurring acid soils and lime-treated (non acid) soils, significant differences were observed for ‘days to flowering’ and ‘plant height,’ but not for ‘number of tillers’ or ‘number of panicles’ (*p* < 0.05). Overall, plants flowered earlier in acid soils than in limed conditions (*p* < 0.05). A stepwise regression analysis identified a region on chromosome 3 that was significantly associated with ‘days to flowering’ under both treatments (LOD > 5.53) and one region on chromosome 9 significantly associated with ‘days to flowering’ under limed soil conditions only (LOD > 5.53; Fig. 6a, b; Online Resource 8a) For plant height, plants under acid soil conditions were, on average, smaller than those under limed conditions. A stepwise regression analysis identified a region on chromosome 10 that was significantly associated (LOD > 3.51) with ‘plant height’ under both treatments, and a region on chromosome 1 significantly associated with ‘plant height’ under natural acid soil conditions (LOD > 3.51; Fig. 6a, b; Online Resource 8b). Based on these examples, we demonstrate that the interspecific ILs developed on this project embody a wide range of phenotypic variation and can be used to dissect the genetics of both simply and quantitatively inherited traits, as well as to broaden the genetic base of elite breeding materials.

## Discussion

We report the development of two sets of interspecific ILs using a drought-tolerant *tropical japonica* cultivar, Curinga (*CUR*), as the recurrent parent and two wild donor accessions from the species *O. meridionalis* (*MER*) and *O. rufipogon* (*RUF*). We demonstrate that the IL populations segregate for traits of interest, and we identified a superior IL, *RUF27,* that has a higher number of tillers and number of panicles than *CUR* under upland soil conditions. This new germplasm offers the rice community an opportunity to explore the genetic potential of variation found in two wild rice species in an elite cultivated background and to identify potentially useful pre-breeding materials.

### Genetic divergence of wild and cultivated materials

Despite the fact that the *O. meridionalis* lineage is estimated to have diverged from the other AA genome species approximately 2 M years ago and remains geographically isolated from both *O. sativa* and *O. rufipogon,* we demonstrate that SSR-based linkage maps were collinear in the two interspecific crosses. This made it possible to use the SSR and indel markers for MABC during three generations of backcrossing to construct the IL populations. Nonetheless, we would expect more structural variation, greater disruption of recombination, and more significant sterility barriers between *MER* and *CUR* than between *RUF* and *CUR.* Consistent with this hypothesis, 26.3 % of the *MER* genome was missing from the IL library reported here, while only 2.4 % of the *RUF* genome was not represented in the *CUR/RUF* population. We also observed fewer and smaller introgressed regions, greater segregation distortion, and had more difficulty making crosses in the *CUR/MER* than in the *CUR/RUF* population. Nevertheless, the theoretical percentage of overall recurrent parent genome expected in a random BC_3_ generation line (93.75 %) was very similar to that obtained in the *CUR/MER* IL library (95 %) reported here.

Linkage analysis identified six regions with strong segregation distortion in the *CUR/MER* map (Online Resource 5**)**. Seven of the fragments that were lost during the development of the *CUR/MER* ILs co-localized with markers showing strong segregation distortion in the BC_1_F_1_ (Online Resource 6a). In addition four of the regions associated with segregation distortion in these materials, on chromosomes 4, 6, 11, and 12 (Online Resource 5), co-localized with previously reported sterility factors (Kinoshita 1991, 1993; Rha et al. 1995; Matsubara and Khin-Thidar 2003). Deviation from expected Mendelian segregation ratios is commonly observed in interspecific crosses of rice (McCouch et al. 1988; Xu et al. 1997; Lorieux et al. 2000; Brondani et al. 2001) and is usually associated with the presence of linked sterility factors and post-zygotic reproductive barriers (Sano 1990; Koide et al. 2008; Garavito et al. 2010; Gutierrez et al. 2010).

It is noteworthy that many of the missing *MER* fragments in the *CUR/MER* ILs were lost during the double haploidization process, rather than during the backcrossing. This suggests a possible sex-dependent transmission-ratio distortion system acting at these loci, such that there was preferential abortion of male gametes possessing the *MER* alleles in favor of those with the *CUR* alleles. Another reason why specific donor segments may be underrepresented or lost from the *CUR/MER* IL population is that they were negatively associated with regeneration ability during the tissue culture process (Xu et al. 1997). Li et al. (2013) identified 25 QTLs associated with tissue culture response from an intraspecific cross between the *indica* variety 93–11 and the *japonica* variety Nipponbare. Many of the QTLs associated with regeneration ability in tissue culture in chromosome 2 (*qCBT*-*2a*, *qCBT*-*2b*, and *qRR*-*7*), and 6 (*qICC*-*6* and *qICF*-*6*) (Li et al. 2013) colocalize with missing regions in the *CUR/MER* ILs.

In contrast to the *CUR/MER* population, segments covering almost the entire *RUF* genome were successfully transmitted to the *CUR/RUF* IL library. However, the recovery of the recurrent parent genome (89.9 %) was lower than expected (93.75 %). This is partly due to the low genomic coverage with SSRs, and possibly because the populations used for selection during the cycles of MABC were not large enough to enable us to identify the most desirable combination of target introgression(s) and clean genetic background.

### Utility of GBS for enhancing marker density

Currently, GBS is being adopted as an alternative to the use of fixed SNP arrays for the generation of high-density marker data in plant breeding and genetics. This is largely due to the simplification and automation of library preparation production protocols, the high throughput and relatively low cost of sequencing, the ability to simultaneously discover and genotype SNPs, and the reduction in ascertainment bias (Heslot et al. 2013). However, the versatility and potential for widespread use of GBS depends upon the bioinformatics tools available to address the relatively high error rate and the data sparsity of GBS. Several bioinformatics pipelines have been developed to overcome these obstacles (Bradbury et al. 2007; Spindel et al. 2013; Sonah et al. 2013). By using GBS, we were able to increase our maker density over 50-fold, compared to a fixed 6K SNP array, and over 600-fold compared to our initial SSR dataset. The ability to rapidly generate high-density SNP datasets in fixed collections of ILs, such as those developed in this study, makes it possible to define the size and positions of both target and background introgressions and to identify small donor introgressions that had been missed using previous, lower density marker datasets.

### Phenotypic variation

Wild species are differentiated from cultivated forms of rice by numerous genetic changes and trait variation, including pigmentation of the pericarp. Most wild species, including both *O. meridionalis* and *O. rufipogon,* have red pericarp, while in *O. sativa* cultivars, the pericarp is white or translucent. The *RC* gene responsible for the change from red to white pericarp is a basic helix-loop-helix transcription factor, and a 14-bp deletion in exon 6 of the *RC* gene is responsible for the loss of pigmentation in the pericarp tissue (Sweeney et al. 2006). The *RC* gene maps within the introgressed region on chromosome 7 that is associated with red pericarp in the *CUR/MER* and *CUR/RUF* populations (Fig. 4). Here, we demonstrated that ILs with colored pericarp carry an introgression from the donor parent at the *RC* locus (Online Resource 7). Genotypic screening using the functional marker in the *RC* gene confirmed that all the lines with colored pericarp carry at least one *wild-type* allele at *RC*, compared with control lines that have white pericarp and are known to carry the 14-bp deletion in Exon 6 of *RC* (Fig. 4b). These results confirmed our mapping results and demonstrated that the colored phenotype for both IL libraries is associated with the *RC* gene. Proanthocyanidins, the red pigment in rice pericarp, have been associated with some nutritional benefits (Ling et al. 2001) that could be exploited by developing niche markets. ILs identified in this study with red pericarp might be of interest for breeding programs interested in developing value-added rice varieties. Some of the red-pericarp ILs identified in this study carry a single, small, well-defined wild introgression at the *RC* locus on chromosome 7, and the genomic composition of the other ILs is close to 97 % of the RP having all of the agronomic traits of *CUR* and only a small introgression that causes the colored pericarp phenotype.

Upland rice is grown in rainfed, naturally well-drained soils without surface water accumulation (Ahmadi et al. 2004). This agro-ecosystem is particularly prone to stresses such as aluminum toxicity, phosphorus deficiency, drought caused by erratic rainfall, rice blast disease and weeds (Ahmadi et al. 2004). The genetic base of upland rice cultivars in Latin America is particularly narrow due to the fact that a small core of adapted progenitors has been used repeatedly in different rice breeding programs (Guimaraes 1993; Guimaraees et al. 1996). It is therefore important to identify novel allelic variation that can improve the genetic pool of upland varieties. To assess the potential for phenotypic variation among the interspecific ILs developed on this project, we evaluated the 48 *CUR/RUF* ILs for four agronomic traits, ‘Days to flowering,’ ‘Plant height,’ ‘Tiller number’ and ‘Panicle number’ under upland soil conditions in Meta-Colombia.

While variation coming from the wild donor was often associated with traits that were not considered favorable in the context of plant improvement, the reverse was also true. Thus, an *O. rufipogon* introgression on the short arm of chromosome 3 was associated with late flowering (Fig. 6a, b), consistent with reports using the same wild donor in the cv Jefferson (*tropical japonica*) genetic background (Xiao et al. 1998; Moncada et al. 2001; Thomson et al. 2003). The *HEADING DATE 9* (*Hd9*) locus, known to be involved in photoperiod sensitivity, is located in this region (Lin et al. 2002). On average the lines with the *RUF* introgression at the *Hd9* locus flowered 15.5 days later than *CUR*. The MADS-box gene *OsSOC1* is located within the *Hd9* interval (chromosome 3: 1,269,856–1,271,783) (Tadege et al. 2003) and is a candidate gene that may be responsible for the late flowering phenotype of ILs carrying the *RUF* allele at this locus. On chromosome 1, a *RUF* introgression increased the height of ILs under natural acid soil conditions. This region co-localizes with the plant height QTLs, *ph1.1* (Moncada et al. 2001) and *ph1.2* (Thomson et al. 2003) (Fig. 6a, b), and contains the *SEMI*-*DWARF1* (*SD1*) gene associated with the green revolution (Sasaki et al. 2002). The identification of *RUF* introgressions associated with late flowering and taller plant height represent throw backs to ancestral traits, while plant breeders have consciously selected for shorter plants with early flowering traits for production in upland ecosystems in South America (Châtel et al. 2008).

On the other hand, a *RUF* introgression on chromosome 10 was associated with reduced plant height under both limed and natural acid soil conditions. This *RUF* allele contributes transgressive variation for shorter stature that may be of interest to breeders interested in selecting for shorter plants. Similarly, the IL *RUF27* produced more tillers and more panicles than the recurrent parent, *CUR,* while it did not differ in days to flowering or plant height. *RUF27* also had the highest grain yield of any of the ILs in this study, though the difference between *RFU27* and *CUR* was not statistically significant. *RUF27* carries six small introgressions on chromosomes 2, 6, 8, 9, 10 and 12 and it will be interesting to continue backcrossing this line to understand the genetics underlying the higher number of tillers and panicles and to validate the value of this IL for breeding purposes. Further, phenotypic analysis is underway on both populations of ILs under a variety of field and controlled environment conditions to better characterize these lines and their utility as pre-breeding materials.

The ILs described here are available for research purposes via a Material Transfer Agreement from CIAT.

## Electronic supplementary material

Below is the link to the electronic supplementary material.

Supplementary material 1 (PDF 479 kb)

Supplementary material 2 (PDF 776 kb)

Supplementary material 3 (PDF 1783 kb)

Supplementary material 4 (PDF 2331 kb)

Supplementary material 5 (PDF 284 kb)

Supplementary material 6 (PDF 106 kb)

Supplementary material 7 (PDF 898 kb)

Supplementary material 8 (PDF 1893 kb)
