# Supplementary material for: Development and GBS-genotyping of introgression lines (ILs) using two wild species of rice, O. meridionalis and O. rufipogon, in a common recurrent parent, O. sativa cv. Curinga
Source: Mol Breed. 2015 Feb 14;35(2):81. doi: 10.1007/s11032-015-0276-7 (PMC4328105; doi:10.1007/s11032-015-0276-7)
Supplement: Supplementary file 1 — Supplementary material 1 (PDF 479 kb) [file 11032_2015_276_MOESM1_ESM.pdf]

Development and GBS-genotyping of Introgression Lines (ILs) using two wild species of rice, *O. meridionalis* and *O. rufipogon*, in a common recurrent parent, *O. sativa* cv. Curinga. *Molecular Breeding*.

Arbelaez J. D., Moreno L. T., Singh N., Tung C.-W., Maron L. G., Ospina Y., Martinez C. P., Grenier C., Lorieux M., McCouch S. Department of Plant Breeding and Genetics, Cornell University, emails: srm4@cornell.edu

Online Resource 1. Marker Assisted Backcrossing pedigree used to develop the ILs.

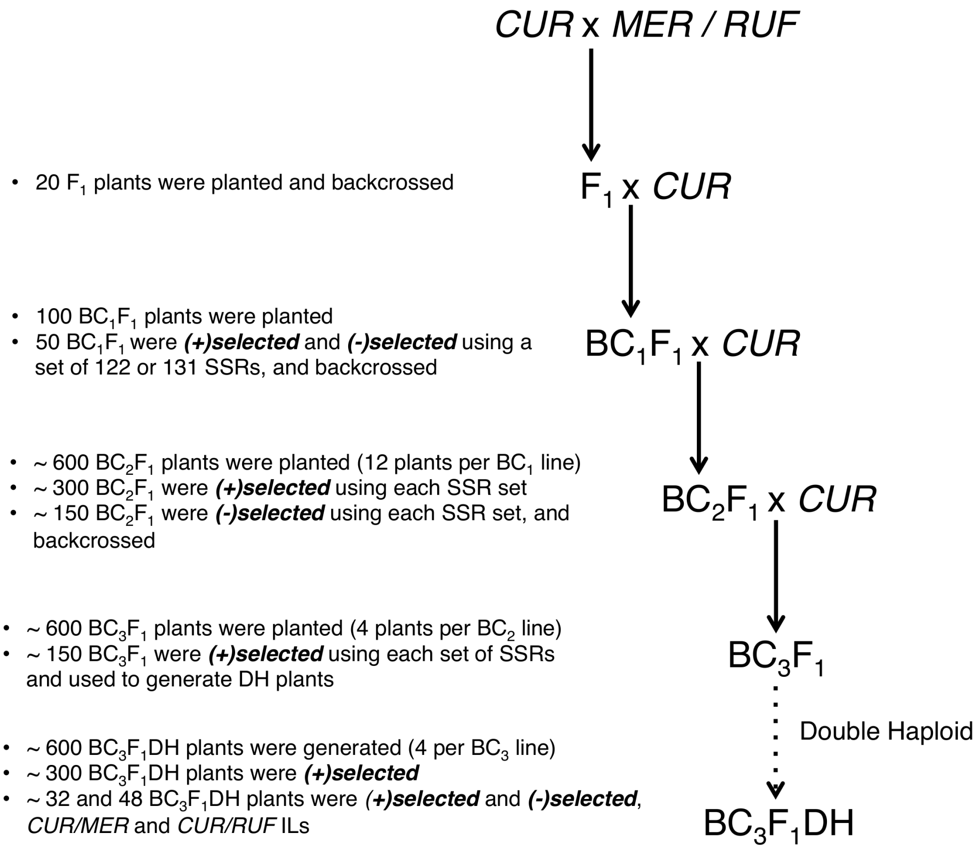

- 20 F_1 plants were planted and backcrossed

- 100 BC_1F_1 plants were planted
- 50 BC_1F_1 were ***(+)selected*** and ***(-)selected*** using a set of 122 or 131 SSRs, and backcrossed

- ~ 600 BC_2F_1 plants were planted (12 plants per BC_1 line)
- ~ 300 BC_2F_1 were ***(+)selected*** using each SSR set
- ~ 150 BC_2F_1 were ***(-)selected*** using each SSR set, and backcrossed

- ~ 600 BC_3F_1 plants were planted (4 plants per BC_2 line)
- ~ 150 BC_3F_1 were ***(+)selected*** using each set of SSRs and used to generate DH plants

- ~ 600 BC_3F_1DH plants were generated (4 per BC_3 line)
- ~ 300 BC_3F_1DH plants were ***(+)selected***
- ~ 32 and 48 BC_3F_1DH plants were ***(+)selected*** and ***(-)selected***, *CUR/MER* and *CUR/RUF* ILs

❖ ***(+)selected*** stands for positive or forward selection that is the selection of a desired donor locus. ***(-)selection*** stands for negative or background selection which is the selection against undesired donor locus.
