# Supplementary material for: Development and GBS-genotyping of introgression lines (ILs) using two wild species of rice, O. meridionalis and O. rufipogon, in a common recurrent parent, O. sativa cv. Curinga
Source: Mol Breed. 2015 Feb 14;35(2):81. doi: 10.1007/s11032-015-0276-7 (PMC4328105; doi:10.1007/s11032-015-0276-7)

Development and GBS-genotyping of Introgression Lines (ILs) using two wild species of rice, *O. meridionalis* and *O. rufipogon*, in a common recurrent parent, *O. sativa* cv. Curinga. *Molecular Breeding*. Arbelaez J. D., Moreno L. T., Singh N., Tung C.-W., Maron L. G., Ospina Y., Martinez C. P., Grenier C., Lorieux M., McCouch S. Department of Plant Breeding and Genetics, Cornell University, emails: srm4@cornell.edu

Online Resource 2. **(a)** Acid-soils field trial soil analysis. For each replication 10 samples were taken and bulked at two different depths, from 0 cm to 10 cm, and from 10 cm to 20 cm. Variables was measured as follows: pH as pH water 1:1, Al (cmol/kg) as exchangeable Al (KCl 1M) vol., P-Brayll (mg/kg) as Bray II phosphorous spectrometry, Ca (cmol/kg) as exchangeable calcium (Ab, At), Mg (cmol/kg) as exchangeable magnesium (Ab, At) and K (cmol/kg) as exchangeable potassium (Ab, At). **(b)** Scatterplot of four agronomical traits for 48 *CUR/RUF* ILs and *CUR* (lower portion from the diagonal), frequency of distribution (diagonal), and correlation values between the 8 traits (upper portion from the diagonal).

a)

Sample	Treatment	Depth	Rep	pH	Al (cmol/kg)	P-BrayII (mg/kg)	Ca (cmol/kg)	Mg (cmol/kg)	K (cmol/kg)	Al-Sat (%)
1	Non-limed	0-10 cm	1	4.63	2.65	13.87	0.59	0.25	0.17	72.45
2	Non-limed	10-20 cm	1	4.62	2.85	4.66	0.53	0.21	0.10	77.22
3	Non-limed	0-10 cm	2	4.52	3.00	14.83	0.56	0.23	0.17	75.63
4	Non-limed	10-20 cm	2	4.45	3.50	3.98	0.34	0.14	0.10	85.82
5	Limed	0-10 cm	1	5.13	1.00	11.58	2.15	0.82	0.16	24.24
6	Limed	10-20 cm	1	4.67	2.50	3.30	0.89	0.38	0.08	64.88
7	Limed	0-10 cm	2	5.36	0.55	11.82	4.12	1.09	0.17	9.27
8	Limed	10-20 cm	2	4.77	1.80	4.74	1.73	0.66	0.09	42.12

b)

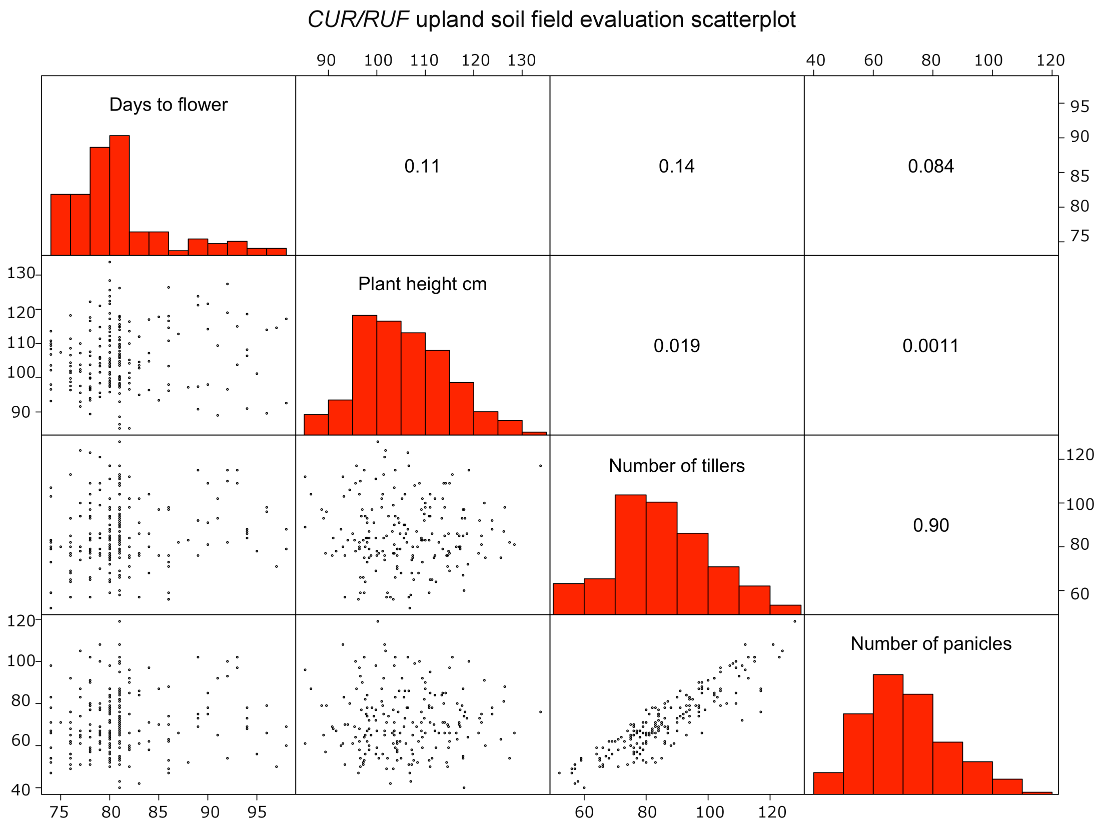

Supplement: Supplementary file 2 — Supplementary material 2 (PDF 776 kb) [file 11032_2015_276_MOESM2_ESM.pdf]
