# Supplementary material for: Development and GBS-genotyping of introgression lines (ILs) using two wild species of rice, O. meridionalis and O. rufipogon, in a common recurrent parent, O. sativa cv. Curinga
Source: Mol Breed. 2015 Feb 14;35(2):81. doi: 10.1007/s11032-015-0276-7 (PMC4328105; doi:10.1007/s11032-015-0276-7)

Development and GBS-genotyping of Introgression Lines (ILs) using two wild species of rice, *O. meridionalis* and *O. rufipogon*, in a common recurrent parent, *O. sativa* cv. Curinga. *Molecular Breeding*. Arbelaez J. D., Moreno L. T., Singh N., Tung C.-W., Maron L. G., Ospina Y., Martinez C. P., Grenier C., Lorieux M., McCouch S. Department of Plant Breeding and Genetics, Cornell University, emails: srm4@cornell.edu

Online Resource 3a. Linkage map developed from 110 BC_1F_1 *CUR/MER* plants genotyped with 122 SSRs (listed on the right side of each bar). Marker order was estimated with a LOD = 3 and r_{max} = 0.3. Distances between markers were calculated using the Kosambi mapping function.
*** Indicates markers with strong segregation distortions.

CUR/MER BC1F1 Linkage map

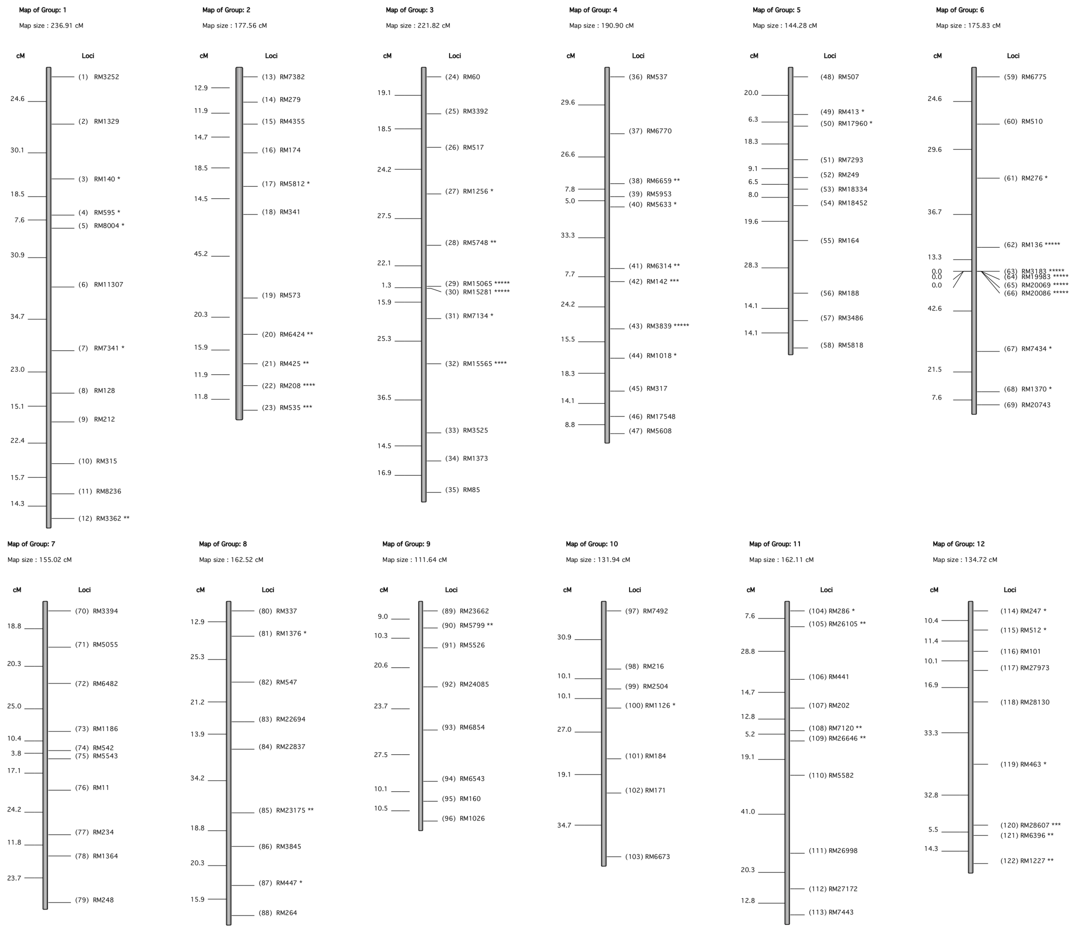

Online Resource 3b. Linkage map developed from 80 BC_1F_1 *CUR/RUF* plants genotyped with 131 SSRs listed on the right side of each bar. Marker order was estimated with a LOD = 3 and r_{max} = 0.3. Distances between markers were calculated using the Kosambi mapping function.

CUR/RUF BC1F1 Linkage map

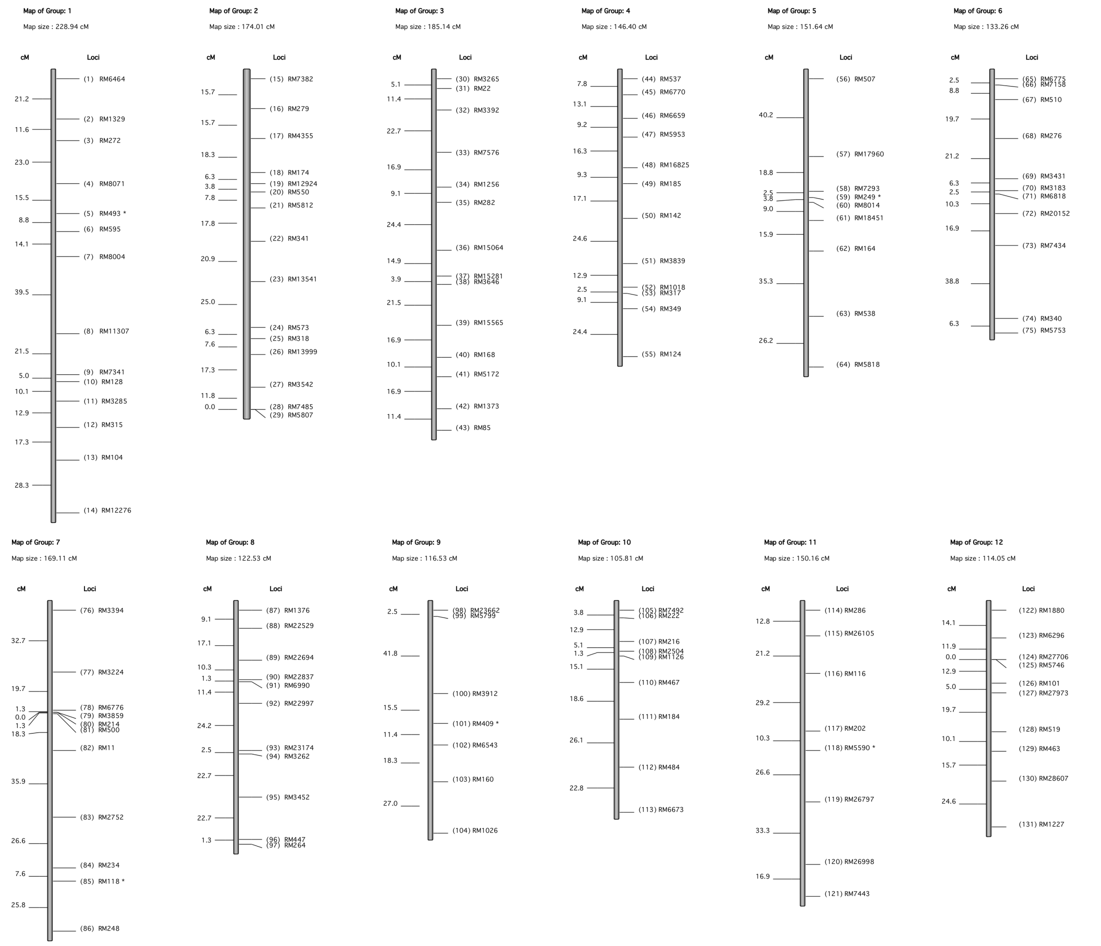

Supplement: Supplementary file 3 — Supplementary material 3 (PDF 1783 kb) [file 11032_2015_276_MOESM3_ESM.pdf]
