# Supplementary material for: Development and GBS-genotyping of introgression lines (ILs) using two wild species of rice, O. meridionalis and O. rufipogon, in a common recurrent parent, O. sativa cv. Curinga
Source: Mol Breed. 2015 Feb 14;35(2):81. doi: 10.1007/s11032-015-0276-7 (PMC4328105; doi:10.1007/s11032-015-0276-7)
Supplement: Supplementary file 4 — Supplementary material 4 (PDF 2331 kb) [file 11032_2015_276_MOESM4_ESM.pdf]

Development and GBS-genotyping of Introgression Lines (ILs) using two wild species of rice, *O. meridionalis* and *O. rufipogon*, in a common recurrent parent, *O. sativa* cv. Curinga. *Molecular Breeding*. Arbelaez J. D., Moreno L. T., Singh N., Tung C.-W., Maron L. G., Ospina Y., Martinez C. P., Grenier C., Lorieux M., McCouch S. Department of Plant Breeding and Genetics, Cornell University, emails: srm4@cornell.edu

Online Resource 4a. Graphical comparison between the physical distances (left bar) estimated in pseudo-cM (base pair distance reported for each SSR, divided by the recombination frequency or average number of bp/cM for each chromosome), versus the genetic distance (right bar) in the BC_1F_1 population derived from *CUR/MER* (**top**). Genetic linkage map summaries for *CUR/MER*. The average distance between markers is expressed as the ratio between the genetic linkage group distances (cM) over the number of markers mapped per chromosome. Difference between genetic and physical distance is the subtraction between the genetic distance and the physical distance (cM) (**bottom**).

CUR/MER physical vs. genetic distance comparison

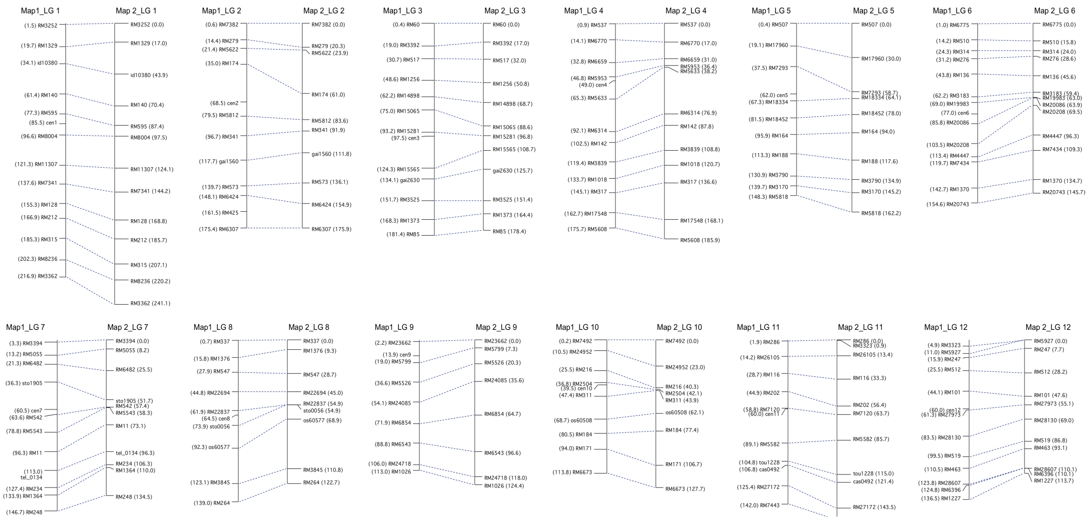

CUR/MER linkage groups					
**Linkage group**	**Number of markers**	**Genetic distance (cM)**	**Average distance between markers (cM)**	**Physical distance (pseudo-cM)**	**Difference between genetic and physical (cM)**
1	12	236.91	19.74	216.9	20.01
2	11	177.56	16.14	175.4	2.16
3	12	221.82	18.49	181.4	40.42
4	12	190.9	15.91	175.µ7	15.2
5	11	144.28	13.12	148.3	-4.02
6	11	175.83	15.98	154.6	21.23
7	10	155.02	15.50	146.7	8.32
8	9	162.52	18.06	139	23.52
9	8	111.64	13.96	113	-1.36
10	7	131.94	18.85	118.8	13.14
11	10	162.11	16.21	142	20.11
12	9	134.72	14.97	136.5	-1.78
**Total**	122	2005.25	16.41	1848.3	156.95

Online Resource 4b. Graphical comparison between the physical distances (left bar) estimated in pseudo-cM (base pair distance reported for each SSR, divided by the recombination frequency or average number of bp/cM for each chromosome), versus the genetic distance (right bar) in the BC_1F_1 population derived from *CUR/RUF* (**top**). Genetic linkage map summaries for *CUR/RUF*. The average distance between markers is expressed as the ratio between the genetic linkage group distances (cM) over the number of markers mapped per chromosome. Difference between genetic and physical distance is the subtraction between the genetic distance and the physical distance (cM) (**bottom**).

CUR/RUF physical vs. genetic distance comparison

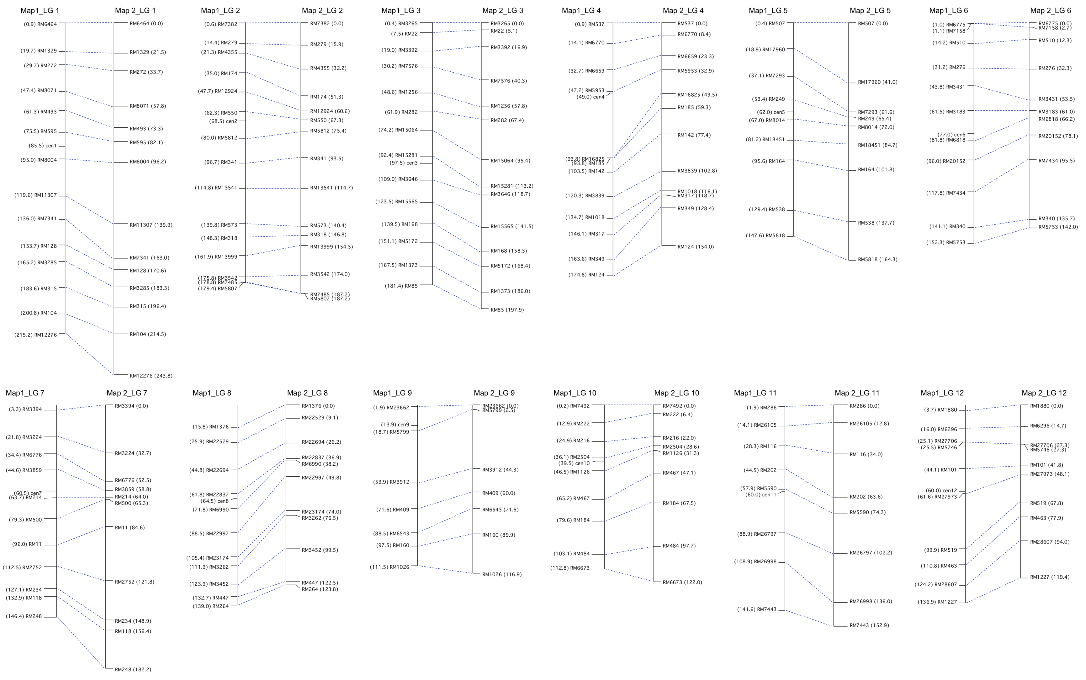

***CUR/RUF* linkage groups**					
**Linkage group**	**Number of markers**	**Genetic distance (cM)**	**Average distance between markers (cM)**	**Physical distance (pseudo-cM)**	**Difference between genetic and physical (cM)**
1	14	228.94	16.35	215.2	13.74
2	15	174.01	11.60	179.4	-5.39
3	14	185.14	13.22	181.4	3.74
4	12	146.4	12.20	174.8	-28.4
5	9	151.64	16.85	147.6	4.04
6	11	133.26	12.11	152.3	-19.04
7	11	169.11	15.37	146.4	22.71
8	11	122.53	11.14	139	-16.47
9	7	116.53	16.65	115.5	1.03
10	9	105.81	11.76	112.8	-6.99
11	8	150.16	18.77	141.6	8.56
12	10	114.05	11.41	136.9	-22.85
**Total**	131	1797.58	13.95	1842.9	-45.32
