# Supplementary material for: Development and GBS-genotyping of introgression lines (ILs) using two wild species of rice, O. meridionalis and O. rufipogon, in a common recurrent parent, O. sativa cv. Curinga
Source: Mol Breed. 2015 Feb 14;35(2):81. doi: 10.1007/s11032-015-0276-7 (PMC4328105; doi:10.1007/s11032-015-0276-7)
Supplement: Supplementary file 5 — Supplementary material 5 (PDF 284 kb) [file 11032_2015_276_MOESM5_ESM.pdf]

Development and GBS-genotyping of Introgression Lines (ILs) using two wild species of rice, *O. meridionalis* and *O. rufipogon*, in a common recurrent parent, *O. sativa* cv. Curinga. *Molecular Breeding*. Arbelaez J. D., Moreno L. T., Singh N., Tung C.-W., Maron L. G., Ospina Y., Martinez C. P., Grenier C., Lorieux M., McCouch S. Department of Plant Breeding and Genetics, Cornell University, emails: srm4@cornell.edu

Online Resource 5. (**a**) Segregation distortion analysis of SSR markers mapped in the BC_1F_1 from *CUR/MER*. Each point represents the percentage deviation with respect of the recurrent genotypic class expected (50 %, solid blue line). The dash black line and the dotted red line represent the critical values for a probability value of 0.5 and 0.01 respectively. (**b**) Markers with strong segregations distortion in the *CUR/MER* BC_1F_1 linkage analysis. The genotypic classes are defined as homozygous *CUR* class (hmz. *CUR*) and heterozygous class (heterozygote). A significance threshold level of 0.01 was set to identify markers with strong deviation from a chi-square test.

a)

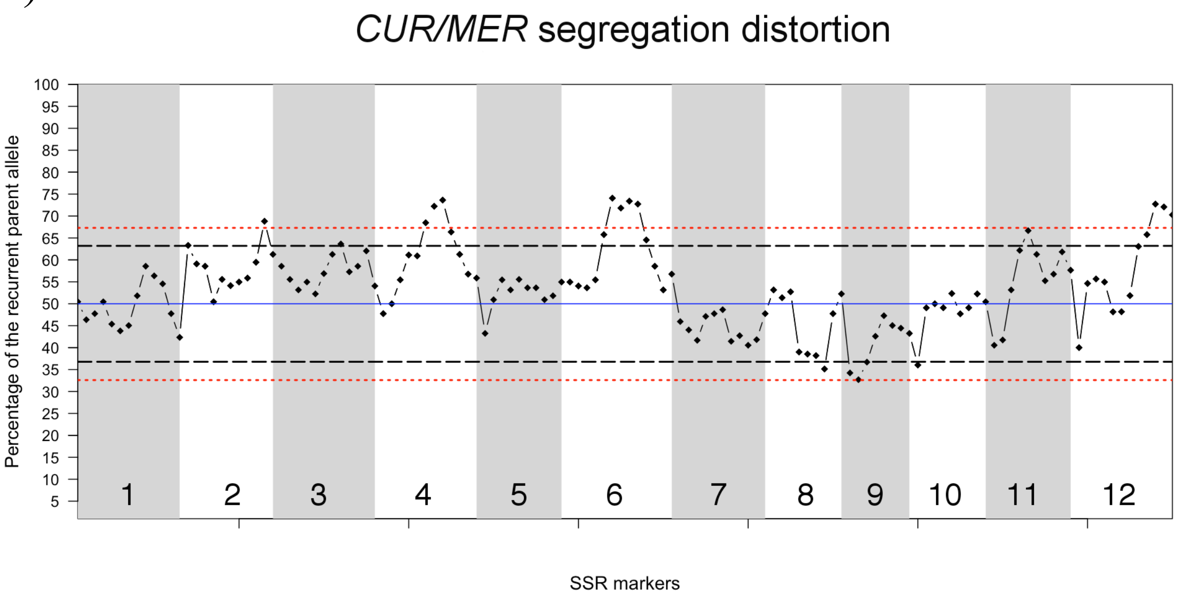

b)

Marker	Chr	Chi-square	p-value	Genotypic hmz. class	Genotypic htz. class	Skewed genotypic class	% hmz.	Associated sterility gene
RM425	2	15.42	0.000086	75	34	hmz. *CUR*	68.81	-
RM6314	4	15.14	0.000100	76	34	hmz. *CUR*	68.47	*s-e-2, ga-6, ga-10**
RM142	4	21.33	0.000004	78	30	hmz. *CUR*	72.22	*s-e-2, ga-6, ga-10**
RM3839	4	24.58	0.000001	81	29	hmz. *CUR*	73.64	*s-e-2, ga-6, ga-10**
RM1018	4	11.78	0.000598	73	37	hmz. *CUR*	66.36	*s-e-2, ga-6, ga-10**
RM136	6	11.04	0.000894	73	37	hmz. *CUR*	65.77	*Su-Cif****
RM3183	6	25.04	0.000001	80	28	hmz. *CUR*	74.07	*Su-Cif****
RM19983	6	20.95	0.000005	79	31	hmz. *CUR*	71.82	*Su-Cif****
RM20086	6	23.86	0.000001	80	29	hmz. *CUR*	73.39	*Su-Cif****
RM20208	6	22.73	0.000002	80	30	hmz. *CUR*	72.73	*Su-Cif****
RM23662	9	11.04	0.000894	38	72	heterozygote	34.23	-
RM5799	9	12.46	0.000416	34	70	heterozygote	32.69	-
RM5526	9	7.72	0.005475	40	69	heterozygote	36.70	-
RM7120	11	12.00	0.000532	72	36	hmz. *CUR*	66.67	*S-3*
RM463	12	11.04	0.000894	72	38	hmz. *CUR*	65.77	*ga-13***
RM28607	12	22.73	0.000002	80	30	hmz. *CUR*	72.73	*ga-13***
RM6396	12	21.63	0.000003	80	30	hmz. *CUR*	72.07	*ga-13***
RM1227	12	18.24	0.000019	78	32	hmz. *CUR*	70.27	*ga-13***

* *ga-6, ga-10, s-e-2* and S-3 genes were listed by Kinoshita (1991, 1993)
** *ga-13* was listed by Rha et al. (1995)
*** The dominant suppressor *Su-Cif* was listed by Matsubara et al. (2003).
