# Supplementary material for: Development and GBS-genotyping of introgression lines (ILs) using two wild species of rice, O. meridionalis and O. rufipogon, in a common recurrent parent, O. sativa cv. Curinga
Source: Mol Breed. 2015 Feb 14;35(2):81. doi: 10.1007/s11032-015-0276-7 (PMC4328105; doi:10.1007/s11032-015-0276-7)
Supplement: Supplementary file 6 — Supplementary material 6 (PDF 106 kb) [file 11032_2015_276_MOESM6_ESM.pdf]

Development and GBS-genotyping of Introgression Lines (ILs) using two wild species of rice, *O. meridionalis* and *O. rufipogon*, in a common recurrent parent, *O. sativa* cv. Curinga. *Molecular Breeding*. Arbelaez J. D., Moreno L. T., Singh N., Tung C.-W., Maron L. G., Ospina Y., Martinez C. P., Grenier C., Lorieux M., McCouch S. Department of Plant Breeding and Genetics, Cornell University, emails: srm4@cornell.edu

Online Resource 6a. Regions with missing introgressions from the donor parent *MER* in the 32 *CUR/MER* ILs. Regions are associated with SNP ID, chromosome, physical position, size, SSRs with strong segregation distortion in the BC_1F_1 generation.

SNP	Chromosome	SNP position bp	Missing segment size bp	Segregation-distorted SSR	SSR position bp
S1_22254822	1	22254822	2340732		
S1_24595554		24595554			
S1_25974004	1	25974004	12672649		
S1_38646653		38646653			
S2_3779	2	3779	15686887		
S2_15690666		15690666			
S2_30577767	2	30577767	3844708	RM425	32297904
S2_34422475		34422475			
S3_2244580	3	2244580	6215963		
S3_8460543		8460543			
S3_9246374	3	9246374	3365607		
S3_12611981		12611981			
S4_21842406	4	21842406	11808013	RM142	20518899
S4_33650419		33650419		RM3839	23904586
				RM1018	26782001
S5_24577616	5	24577616	3268973		
S5_27846589		27846589			
S5_29808179	5	29808179	116873		
S5_29925052		29925052			
S6_7498156	6	7498156	20072852	RM132	8751256
S6_27571008		27571008		RM3183	12446983
				RM19983	13808188
S6_27595529	6	27595529	3622109	RM20086	17163417
S6_31217638		31217638		RM20208	20705943
S7_26127859	7	26127859	3552099		
S7_29679958		29679958			
S10_11545597	10	11545597	3213214		
S10_14758811		14758811			
S12_22673430	12	22673430	1812903	RM463	22091967
S12_24486333		24486333		RM28607	24766329
S12_24557197	12	24557197	2967692	RM6396	24966349
S12_27524889		27524889		RM1227	27305234

Online Resource 6b. Regions with missing introgressions from the donor parent *RUF* in the 48 *CUR/RUF* ILs.

SNP	Chromosome	SNP position bp	Missing segment size bp
S4_28562666	4	28562666	217827
S4_28780493	4	28780493	
S4_28877188	4	28877188	521375
S4_29398563	4	29398563	
S4_29487221	4	29487221	1642898
S4_31130119	4	31130119	
S6_26578765	6	26578765	242265
S6_26821030	6	26821030	
S6_28009274	6	28009274	345620
S6_28354894	6	28354894	
S6_28458078	6	28458078	1918849
S6_30376927	6	30376927	
S6_30762479	6	30762479	442016
S6_31204495	6	31204495	
S8_4308627	8	4308627	2518103
S8_6826730	8	6826730	
S8_7228778	8	7228778	947537
S8_8176315	8	8176315	
S8_8328655	8	8328655	120054
S8_8448709	8	8448709	
S12_19434483	12	19434483	68516
S12_19502999	12	19502999	
