# Supplementary material for: Development and GBS-genotyping of introgression lines (ILs) using two wild species of rice, O. meridionalis and O. rufipogon, in a common recurrent parent, O. sativa cv. Curinga
Source: Mol Breed. 2015 Feb 14;35(2):81. doi: 10.1007/s11032-015-0276-7 (PMC4328105; doi:10.1007/s11032-015-0276-7)

Development and GBS-genotyping of Introgression Lines (ILs) using two wild species of rice, *O. meridionalis* and *O. rufipogon*, in a common recurrent parent, *O. sativa* cv. Curinga. *Molecular Breeding*. Arbelaez J. D., Moreno L. T., Singh N., Tung C.-W., Maron L. G., Ospina Y., Martinez C. P., Grenier C., Lorieux M., McCouch S. Department of Plant Breeding and Genetics, Cornell University, emails: srm4@cornell.edu

Online Resource 7. (**a**) Pericarp color evaluation and genotypic evaluation for the RP *CUR* the two donor parents *MER* and *RUF*, 17 ILs and two control lines (cv IR64 and Azucena). (**b**) Pericarp color of the recurrent parent *CUR* and of the donor parents *RUF* and *MER*. Pericarp color for six ILs with different allele combination for the loci *Rd* and *Rc*. The – sign represent the recurrent parent allele *CUR* and the + sign represent the presence of the donor allele *MER* or *RUF* based on the GBS genotypic data

a)

Genotype	***Rc*** **loci donor introgression**	***Rd*** **loci donor introgression**	**Color phenotype**	**14-bp length** ***Rc*** **deletion**
CUR	-	-	*White*	*Yes*
MER	+	+	*Red*	*No*
MER16	+	-	*Red*	*No*
MER23	+	-	*Red*	*No*
MER22	-	+	*White*	*Yes*
MER24	-	-	*White*	*Yes*
MER15	-	-	*White*	*Yes*
MER17	-	-	*White*	*Yes*
RUF	+	+	*Red*	*No*
RUF01	+	-	*Red*	*No*
RUF04	+	+	*Red*	*No*
RUF07	-	+	*White*	*Yes*
RUF08	+	+	*Red*	*No*
RUF10	-	+	*White*	*Yes*
RUF12	-	+	*White*	*Yes*
RUF29	+	-	*Red*	*No*
RUF32	-	+	*White*	*Yes*
RUF36	+	+	*Red*	*No*
RUF39	+	-	*Red*	*No*
RUF41	+	+	*Red*	*No*
IR64	-	-	*White*	*Yes*
Azucena	-	-	*White*	*Yes*

b)

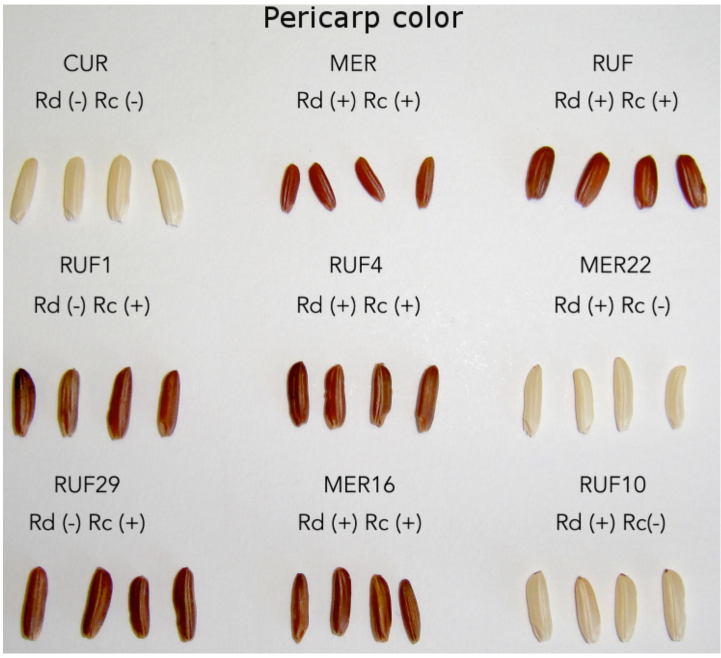

Supplement: Supplementary file 7 — Supplementary material 7 (PDF 898 kb) [file 11032_2015_276_MOESM7_ESM.pdf]
