# Supplementary material for: Development and GBS-genotyping of introgression lines (ILs) using two wild species of rice, O. meridionalis and O. rufipogon, in a common recurrent parent, O. sativa cv. Curinga
Source: Mol Breed. 2015 Feb 14;35(2):81. doi: 10.1007/s11032-015-0276-7 (PMC4328105; doi:10.1007/s11032-015-0276-7)

Development and GBS-genotyping of Introgression Lines (ILs) using two wild species of rice, *O. meridionalis* and *O. rufipogon*, in a common recurrent parent, *O. sativa* cv. Curinga. *Molecular Breeding*. Arbelaez J. D., Moreno L. T., Singh N., Tung C.-W., Maron L. G., Ospina Y., Martinez C. P., Grenier C., Lorieux M., McCouch S. Department of Plant Breeding and Genetics, Cornell University, emails: srm4@cornell.edu

Online Resource 8. Stepwise Regression Single Marker Analysis (SR-SMA) in *CUR/RUF* for (**a**)"Days to flowering" and (**b**) "Average plant height" (**c**) "Number of tillers", and (**d**) "Number of panicles" evaluated under natural acid conditions (Environment 1), and limed conditions (Environment 2). Bar graphs show LOD values for each marker; significance thresholds (horizontal red line) estimated from 1000 permutations corresponding to experiment-wise α = 0.05. (**a**) "Days to flowering"; threshold LOD = 5.5, (**b**) "Average plant height"; threshold LOD = 3.5. For (**c**, **d**) "Number of tillers", and "Number of panicles"; threshold LOD = 3.0 (Note, significance threshold not shown because its value surpassed the scale of the LOD axis on the graph; no QTLs detected for these traits above LOD = 3.0).

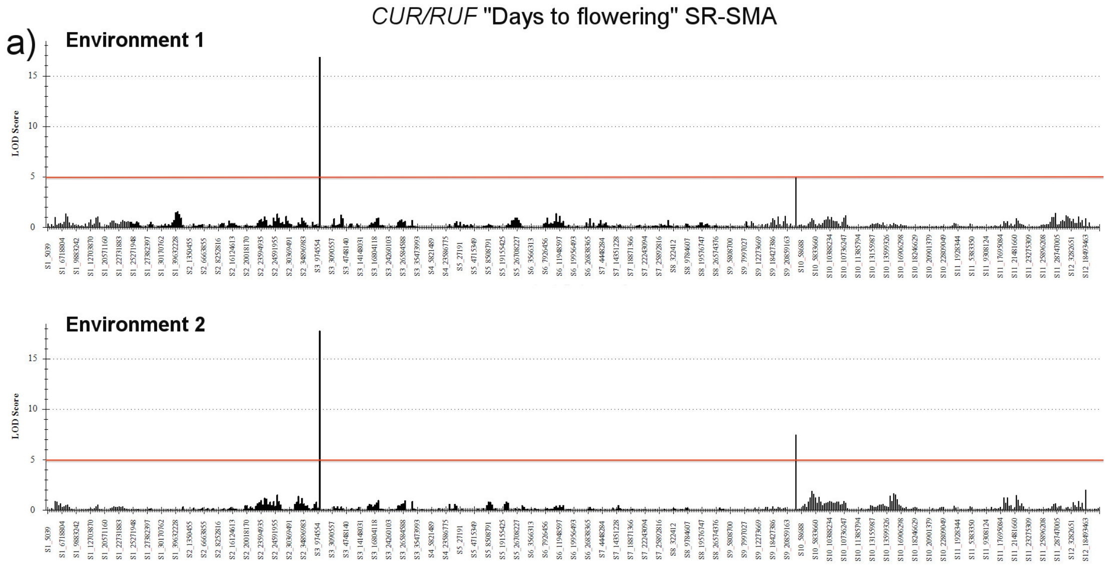

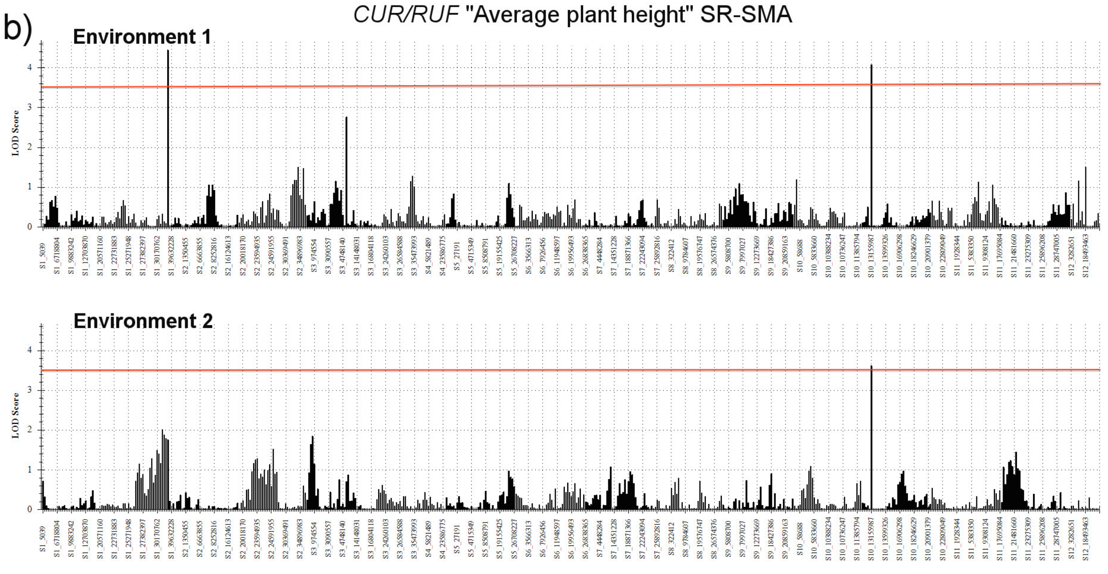

c) *CUR/RUF* "Number of tillers" SR-SMA

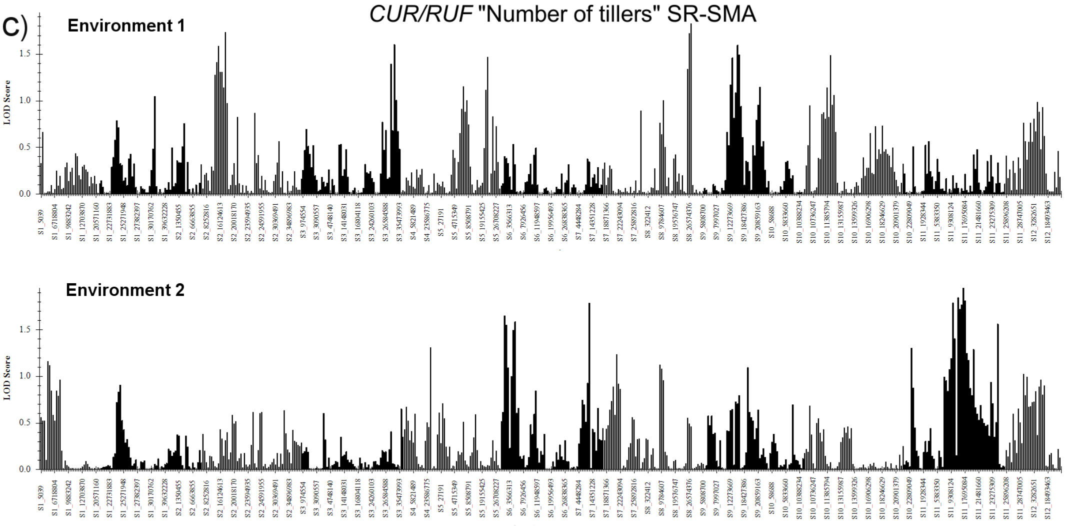

d) *CUR/RUF* "Number of panicles" SR-SMA

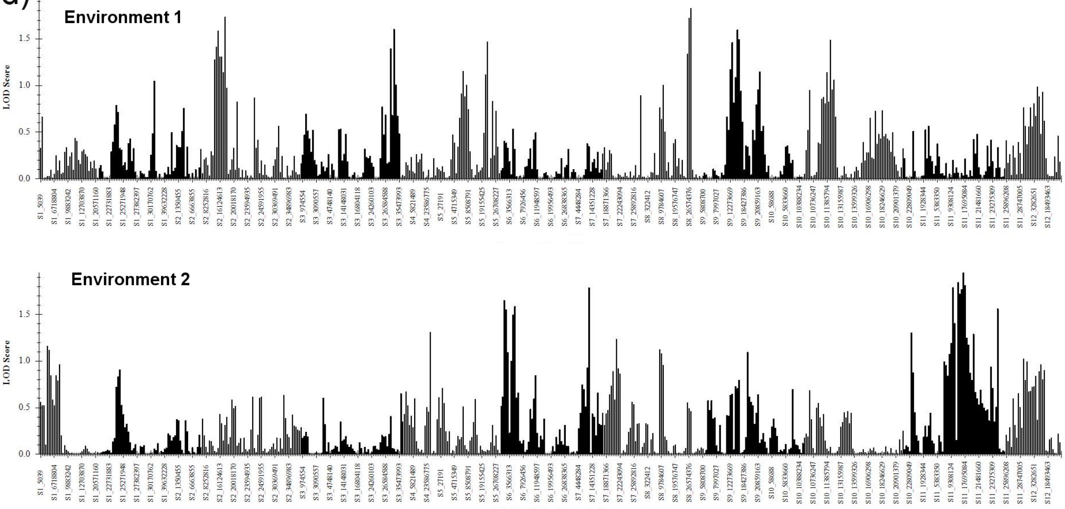

Supplement: Supplementary file 8 — Supplementary material 8 (PDF 1893 kb) [file 11032_2015_276_MOESM8_ESM.pdf]
